# ADSCs stimulated by VEGF-C alleviate intestinal inflammation via dual mechanisms of enhancing lymphatic drainage by a VEGF-C/VEGFR-3-dependent mechanism and inhibiting the NF-*κ*B pathway by the secretome

**DOI:** 10.1186/s13287-022-03132-3

**Published:** 2022-09-05

**Authors:** Lei Zhang, Chen Ye, Peng Li, Chuanding Li, Weigang Shu, Yujie Zhao, Xiaolei Wang

**Affiliations:** 1 Department of Gastroenterology, Shanghai Tenth People’s Hospital, Tongji University School of Medicine, Shanghai, 200072 China; 2 Medical College of Soochow University, Suzhou, 215000 Jiangsu Province China; 3 Shanghai Institute for Advanced Immunochemical Studies, ShanghaiTech University, Shanghai, 201210 China

**Keywords:** Adipose derived-stem cells, Vascular endothelial growth factor C, Chronic colitis, Lymphatic drainage, Paracrine, Faecal microbiota

## Abstract

**Background:**

Adipose-derived stem cells (ADSCs) have provided promising applications for Crohn’s disease (CD). However, the practical efficacy of ADSCs remains controversial, and their mechanism is still unclear. Based on the pathogenesis of dysregulated immune responses and abnormal lymphatic alterations in CD, vascular endothelial growth factor-C (VEGF-C) is thought to be a favourable growth factor to optimize ADSCs. We aimed to investigate the efficacy of VEGF-C-stimulated ADSCs and their dual mechanisms in both inhibiting inflammation “IN” and promoting inflammation “OUT” in the intestine.

**Methods:**

Human stem cells isolated from adipose tissues were identified, pretreated with or without 100 ng/ml VEGF-C and analysed for the secretion of cell culture supernatants in vitro. Lymphatic endothelial cells (LECs) were treated with ADSCs-conditioned medium or co-cultured with ADSCs and VEGF-C stimulated ADSCs. Changes in LECs transmigration, and VEGF-C/VEGFR-3 mRNA levels were assessed by transwell chamber assay and qRT–PCR. ADSCs and VEGF-C-stimulated ADSCs were intraperitoneally injected into mice with TNBS-induced chronic colitis. ADSCs homing and lymphatic vessel density (LVD) were evaluated by immunofluorescence staining. Lymphatic drainage was assessed using Evans blue. Cytokines and growth factors expression was detected respectively by ELISA and qRT–PCR. The protein levels of VEGF-C/VEGFR-3-mediated downstream signals and the NF-*κ*B pathway were assayed by western blot. Faecal microbiota was measured by 16S rRNA sequencing.

**Results:**

ADSCs stimulated with VEGF-C released higher levels of growth factors (VEGF-C, TGF-β1, and FGF-2) and lower expression of cytokines (IFN-γ and IL-6) in cell supernatants than ADSCs in vitro (all *P* < 0.05). Secretome released by VEGF-C stimulated ADSCs exhibited a stronger LEC migratory capability and led to elevated VEGF-C/VEGFR-3 expression, but these effects were markedly attenuated by VEGFR-3 inhibitor. VEGF-C-stimulated ADSCs homing to the inflamed colon and mesenteric lymph nodes (MLNs) can exert stronger efficacy in improving colitis symptoms, reducing inflammatory cell infiltration, and significantly enhancing lymphatic drainage. The mRNA levels and protein concentrations of anti-inflammatory cytokines and growth factors were markedly increased with decreased proinflammatory cytokines in the mice treated with VEGF-C-stimulated ADSCs. Systemic administration of VEGF-C-stimulated ADSCs upregulated the colonic VEGF-C/VEGFR-3 pathway and activated downstream AKT and ERK phosphorylation signalling, accompanied by decreased NF-*κ*B p65 expression. A higher abundance of faecal *p-Bacteroidetes* and lower *p-Firmicutes* were detected in mice treated with VEGF-C-stimulated ADSCs (all *P* < 0.05).

**Conclusion:**

VEGF-C-stimulated ADSCs improve chronic intestinal inflammation by promoting lymphatic drainage and enhancing paracrine signalling via activation of VEGF-C/VEGFR-3-mediated signalling and inhibition of the NF-*κ*B pathway. Our study may provide a new insight into optimizing ADSCs treatment and investigating potential mechanisms in CD.

**Supplementary Information:**

The online version contains supplementary material available at 10.1186/s13287-022-03132-3.

## Background

Crohn’s disease (CD) is a chronic inflammatory disease characterized by an aberrant mucosal immune response in the digestive tract, occurring in genetically susceptible individuals and with the participation of the gut microbiome [1]. To date, the exact aetiology of CD is unknown, and therapies are limited. Although it has entered the era of biological agents, some patients still lack useful treatments due to the secondary failure of drugs and intolerance of adverse effects [2, 3]. Recently, multiple clinical trials have shown that mesenchymal stem cells (MSCs) are effective for the local treatment of complex CD anal fistulas [4, 5]. However, the efficacy and mechanisms of systematic MSCs therapy in luminal CD still require further research [6].

As a type of adult stem cell with multilineage differentiation potential and self-renewal properties, MSCs exert anti-inflammatory and immunosuppressive effects via two main mechanisms, including direct interaction with multiple cell types (including immune cells, endothelial cells, preadipocytes and pericytes) and indirect paracrine effects through secreting numerous soluble mediators (VEGFs, FGFs, TGF-β1 and IL-10) and exosomes [7, 8]. Because of their abundant sources and stable biological properties, adipose-derived stem cells (ADSCs) have been successfully applied in various inflammatory and autoimmune diseases, including osteoarthritis, inflammatory bowel disease (IBD) and graft-versus-host disease (GVHD) [8, 9]. Nonetheless, the clinical efficacy of ADSCs in treating CD is inconsistent. To optimize the therapeutic effects of ADSCs, many efforts have been made in recent in vitro studies. The most concerning are 3D culture, hypoxia, genetic engineering, cytokine pretreatment, etc. [10, 11]. Previous studies have verified that stem cell preconditioning could dynamically alter the composition of their secretome. Vascular endothelial growth factor (VEGF) family members play a pivotal role in regulating the differentiation of MSCs to chondrogenesis, endothelial cells and myoblasts [12–14] and inducing angiogenesis and lymphangiogenesis to enhance the therapeutic outcomes of MSCs [15, 16]. Their secretion by MSCs was obviously elevated in culture systems with inflammatory cytokine stimulation, hypoxia, or 3D culture [17–19]. However, the mechanisms of VEGFs on MSCs in regulating intestinal inflammation have not been elucidated.

In addition to the “IN” of chronic inflammation (the mechanisms of immunoinflammatory cell infiltration, bacterial and foreign antigen invasion, angiogenesis via intestinal microvasculature), the “OUT” of chronic inflammation (the lymphatics and their functions) is another new perspective to control inflammatory bowel disease (IBD). The intestinal lymphatic vascular system serves as an essential modality in resolving tissue oedema, leucocyte transportation, inflammatory chemokines and bacterial clearance [20, 21]. Increased pathological hyperplasia of lymphatic vessels and lymphatic vascular dysfunction are visible in active CD [22]. The main molecular mechanism that drives lymphangiogenesis is the VEGF-C/VEGFR-3 signalling axis. When VEGF-C binds to VEGFR-3 on lymphatic endothelial cells (LECs), numerous downstream pathways associated with lymphangiogenesis are activated, including AKT and ERK [23–25].

VEGF-C has been reported to be not only the most potent lymphatic growth factor for promoting lymphatic formation and maintaining the normal structure and function of intestinal lymphatics [26] but also a main paracrine factor secreted by ADSCs. Our previous studies have indicated that VEGF-C enhances lymphatic drainage to promote inflammation “OUT” of the intestine and then relieves chronic colitis [27]. In addition, ADSCs stimulated by VEGF-C were reported to have stronger proliferation and survival abilities. ADSCs with high expression of VEGF-C and lymphatic endothelial markers (podoplanin and Prox-1) further promoted lymphangiogenesis in vitro [16]. Therefore, therapeutic strategies based on enhancing lymphatic drainage are supposed to provide a new perspective on relieving intestinal inflammation. This optimized treatment of ADSCs may improve the efficacy of ADSCs treatment and have potential applications. We hypothesized that ADSCs stimulated by VEGF-C could play dual effects to alleviate colitis, including enhancing paracrine function to strengthen anti-inflammatory and immunomodulatory roles and increasing lymphatic drainage to promote inflammation out of the gut.

In this study, we confirmed that ADSCs pretreated with VEGF-C maintained the characteristics of MSCs in vitro, promoted the paracrine activity of ADSCs and altered the compositions of secretome comprising growth factors and cytokines. In vivo, VEGF-C-stimulated ADSCs activated the VEGF-C/VEGFR-3 signalling pathway and further upregulated downstream AKT and ERK signalling to enhance lymphatic drainage and restore homeostasis of the faecal microbiota. Meanwhile, the enhanced secretome inhibited the NF-*κ*B pathway and decreased intestinal inflammation. The novel dual mechanism of VEGF-C-stimulated ADSCs in the inhibition of inflammation “IN” and promotion of inflammation “OUT” of the intestine shed new light on ADSCs application and inflammation-immunity-microbiota mechanisms in chronic colitis.

## Materials and method

### Isolation, culture and pre-conditioning of ADSCs

Ten grams of adipose tissue was collected from an anonymized patient of abdominal subcutaneous discarded tissues after informed consent was obtained. The Ethics Committee of Shanghai Tenth People’s Hospital approved this study. The collected adipose tissue was minced, washed 3 times with phosphate-buffered saline (PBS) and digested with collagenase. An equal volume of complete medium containing mesenchymal stem cell basal medium (MSCBM; Dakewe Biotech, Beijing, China) supplemented with 5% EliteGro™-Adv (EliteCell, Woodway, WI, USA) terminated the digestion. After the digested mixture was filtered and centrifuged, the resuspended cell pellet was seeded in a T25 cm^2^ culture flask. When the primary culture growth fusion reached 80%–90%, cells were digested and inoculated into new flasks for continuous amplification culture.

Cells at passage 5 were stimulated in complete medium supplemented with recombinant human VEGF-C protein (100 ng/ml; Abcam, Cambridge, UK) for 48 h prior to implantation in vivo [16]. ADSCs between passages 3 and 5 were utilized in subsequent experiments in vitro.

### Immunophenotypic characterization and differentiation assay of ADSCs

To identify the ADSCs phenotypic profile before and after VEGF-C stimulation, PE-conjugated anti-human CD29, CD34, CD45, FITC-conjugated anti-human CD44 and APC-conjugated anti-human CD90 antibodies were used (BioLegend, San Diego, CA, USA). Cell aliquots (1 × 10^6^) were incubated with antibodies at a concentration of 2 µg/mL at 4 °C for 30 min. One tube of unstained cells was prepared as a control for the antibodies. The labelled cells were subsequently examined by a BD FACSCalibur flow cytometer (Becton Dickinson Biosciences, San Jose, CA, USA), and data were analysed by FlowJo software (Tree Star, Ashland, OR, USA).

To further examine the multipotency of ADSCs in osteogenesis and adipogenesis, 3 × 10^5^ isolated ADSCs were plated in osteogenic conditioned medium (complete medium supplemented with β-glycerol phosphate, ascorbic acid and dexamethasone) for 14 days, fixed in 4% paraformaldehyde and stained with Alizarin Red. Adipogenesis assays were performed in adipogenic medium (complete medium containing indomethacin, dexamethasone, insulin and 3-isobutyl-l-methyl-xanthine) for 10 days and identified using Oil Red O. All chemicals were purchased from Sigma (St. Louis, MO, USA).

### Migration assay

Transwell chambers with a diameter of 6.5 mm and 8.0 μm pore size membrane (Corning, NY, USA) were used. Briefly, the lower chambers were added with 600 μL of ADSC-Conditioned Medium (ADSC-CM), resuspended ADSCs or 100 ng/mL VEGF-C pre-treated ADSCs. Then, 2 × 10^4^ LECs suspended in 100 μL of ECBM (ScienCell, CA, USA) were seeded in the upper chambers, half of LECs were incubated with VEGFR-3 inhibitor 100 nM SAR131675 (MCE, NJ, USA). After 12 h incubation at 37 °C, the cells on the upper surface of the membrane were removed using a cotton swab. The cells on the lower surface of the filter were fixed and stained using 0.1% crystal violet. Images of the transmigrated LEC were captured at 100 × magnification in 3 random fields.

### ELISA on the culture supernatants of ADSCs

To evaluate the proteins secreted by ADSCs, the concentrations of VEGF-C, TGF-β1, IGF-1, FGF-2, IFN-γ, TNF-α, IL-6 and Il-10 in the cell culture supernatants were analysed using enzyme-linked immunosorbent assay (ELISA) kits (CUSABIO Life Science, Wuhan, China) following the manufacturer’s instructions. The level of each factor was determined by calculating its absorbance at 450 nm using a microplate reader.

### Animals and ethics

Thirty-six female BALB/c mice (4–5 weeks) with a body weight of 16–19 g were purchased from Shanghai SLAC Laboratory Animal Co., Ltd. The mice were housed in a second-class animal room at the Laboratory Animal Center of Tongji University (Shanghai, China) under a standard 12 h light/12 h dark cycle. They were allowed to access sterilized food and water freely. All animal experiments were performed in accordance with the Guidelines for the Care and Use of Laboratory Animals. The experimental procedures were approved by the Ethics Committee of Shanghai Tongji Hospital (KYSB-2016-35).

### Induction and intervention of chronic TNBS colitis

**Fig. 1:**
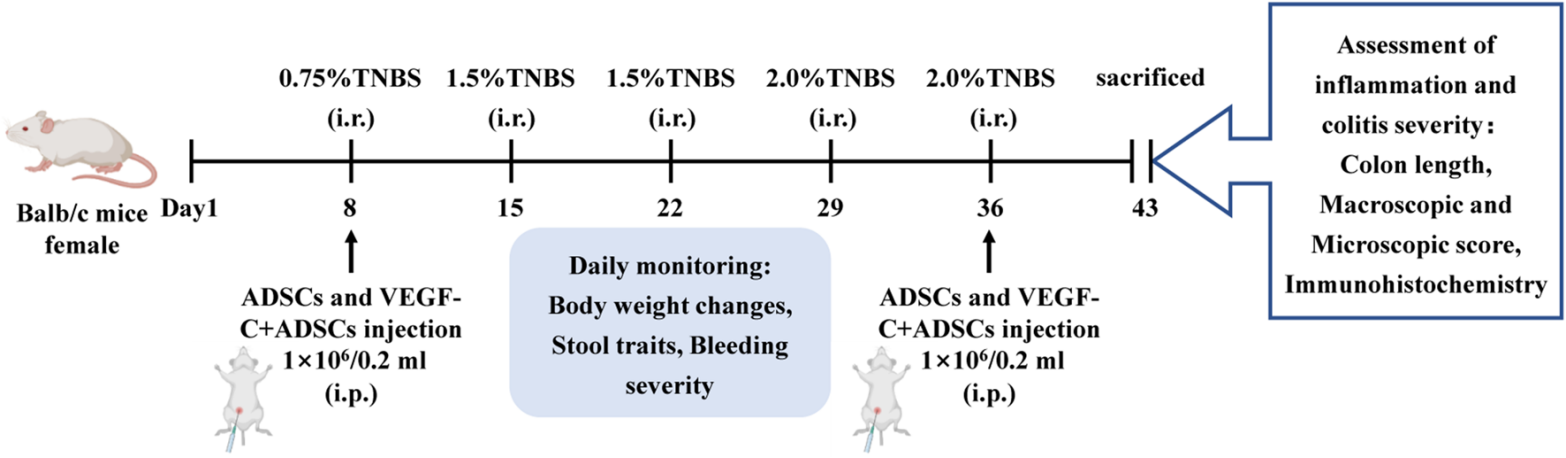
Schematic drawing of colitis establishment, intervention and evaluation in TNBS-induced chronic colitis. Female BALB/c mice at 4–5 weeks of age (*N* = 9 per group) were infused with TNBS into their colons 5 times, and ADSCs and VEGF-C-stimulated ADSCs (1 × 10^6^/0.2 ml) were injected intraperitoneally on Day 8 and Day 36

2,4,6-Trinitrobenzene sulfonic acid (TNBS)-induced chronic colitis was established according to a previously published protocol [28]. In our pre-experiment, we used both female and male 5–6 weeks old mice (each gender 3 mice) to establish experimental colitis, but the male ones often fought at 8–10 weeks of age. The similar situation was also observed in Anje et al., who considered fight could lead to unwanted induction of systemic inflammatory responses [29]. Thus, we chose female mice to perform further experiments. Overall, TNBS experiments were performed on three independent occasions. In this study, mice were weighed and randomly divided into four groups (*N* = 9): (1) control group, (2) TNBS group, (3) ADSCs group, and (4) VEGF-C + ADSCs group. On each 7th day over the next 5 weeks, all mice except the control mice were anaesthetized with 4% chloral hydrate (0.01 ml/g body weight) by intraperitoneal (i.p.) injection and given 100 μL 0.75%, 1.5%, 1.5%, 2.0% and 2.0% TNBS solution (Sigma–Aldrich, St. Louis, MO, USA) in sequence by using a catheter through the anus (Fig. 1).

On Day 8 and Day 36, mice in the ADSCs group and VEGF-C + ADSCs group were injected (i.p.) with 1 × 10^6^ stem cells in 200 μL sterile normal saline (NS) 2 h after TNBS administration. The mice in the TNBS group were injected with the same volume of NS.

### PKH67 staining

To confirm the distribution and homing of injected ADSCs to the colon and mesenteric lymph nodes (MLNs), ADSCs were labelled with a PKH67 fluorescent probe (LMAIbio, Shanghai, China) according to the manufacturer’s guidelines and injected into the abdominal cavity on Day 36. Five days after injection, mice were sacrificed. The tissues were fixed in 4% paraformaldehyde for 48 h to make cryosections for examination. Green fluorescence on PKH67-labelled ADSCs membranes was detected under a fluorescence microscope (Leica Microsystems, Wetzlar, Germany).

### Evaluation of colitis severity

The general status of each mouse, including body weight changes, stool traits and bleeding severity, was observed daily. The colitis score was recorded weekly (Additional file 1: Table 1). The disease activity index (DAI) was determined based on changes in weight loss, faecal status and haematochezia (Additional file 1: Table 2) [30]. The entire colon from the rectum to the caecum was excised, and colon length was measured. Macroscopic lesions of each isolated colon were evaluated by previously described scoring systems (Additional file 1: Table 3) [31, 32]. Microscopic damage was assessed by light microscopy at ×200 magnification with haematoxylin and eosin (H&E) staining. The histological criteria included leukocyte infiltration, bowel wall thickening, crypt structural changes and ulcerations (Additional file 1: Table 4) [33]. All the scores were recorded and assessed by a blind method. Each experiment was repeated three times.

### Immunohistochemical (IHC) analysis

For IHC staining, paraffin-embedded sections of the colons, MLNs and spleens were obtained (5-mm thickness) from each sample. After rehydration with 80% methanol, PBS and PBS with 12% bovine serum albumin (BSA), the sections were incubated with primary antibodies overnight at 4 °C. The next day, the slides were washed with TBS-Tween and incubated with secondary antibodies. Standard H&E staining and immunohistochemical staining were performed as described previously [34]. Primary antibodies were used as follows: CD4 (1: 200), CD8 (1: 300). All antibodies were purchased from Servicebio (Wuhan, China). Additionally, Masson’s trichrome staining was performed using a special kit (Abcam, Cambridge, UK) according to the manufacturer’s instructions. The positively stained cells and collagen/area ratio were counted in five randomly selected fields at a magnification of 200× or 400× using ImageJ software (National Institutes of Health, Bethesda, MD, USA).

### Immunofluorescence staining

Immunofluorescence staining of colonic tissues was performed as previously described [34]. The lymphatic vessels were detected by anti-LYVE-1 (1:100, Abcam, Cambridge, UK)-positive structures with the lumina, and the blood vessels were labelled by anti-CD31 (1:100, Servicebio, Wuhan, China)-positive structures with the lumina. The lymphatic vessel density (LVD, per mm^2^) and microvascular density (MVD, per mm^2^) were calculated in six random fields with the highest density (“hot areas”) of LYVE-1^+^ and CD31^+^ vessels in the mucosa and submucosa by light microscopy at 200 × magnification, respectively. In addition, immunofluorescence staining was also performed to evaluate the dendritic cells (DCs) using CD11c antibody (Invitrogen, CA, USA) in the colon tissues. The positive cells were counted in five randomly selected fields at a magnification of 200× using ImageJ software (National Institutes of Health, Bethesda, MD, USA).

### Lymphatic drainage assay in vivo by Evans blue

Ten micrograms (1%) of Evans blue dye (Sigma–Aldrich, St. Louis, MO, USA) in 10 μL of PBS was injected into the rectal mucosa of anaesthetized mice (*N* = 3 per group) using a Hamilton syringe according to previous studies [35]. Mice were sacrificed after 16 h. Evans blue dye was extracted from the distal colon tissues by incubating at 55 °C overnight in 1 ml formamide (Sangon Biotech, Shanghai, China). The background-subtracted absorbance at 630 nm was measured with a microplate reader (Biotek, Vermont, USA). The concentration of extracted dye was calculated using a standard curve and presented as the absolute amount of dye remaining in the colons.

### Quantitative real-time polymerase chain reaction (qRT–PCR)

Total RNA was extracted from the LECs in the transwell co-culture system and colon tissues using RNA Purification Kit PLUS (EZ-Bioscience, Roseville, USA) and subsequently reverse transcribed to cDNA by PrimeScript™ RT reagent kit (Takara, Ostu, Shiga, Japan) according to the manufacturer’s instructions. qRT–PCR was carried out via a 7900HT Fast real-time PCR system (Applied Biosystems, Foster City, CA, USA) in conjunction with TB Green Premix Ex Taq™ II (Takara, Ostu, Shiga, Japan). The relative mRNA expression levels were evaluated by the 2^−ΔΔCT^ method and normalized to GAPDH expression. Sequences of PCR primers in this study are listed in Additional file 1: Table 5.

### Cytokine immunoassay

The colonic tissues were weighed and homogenized. Cytokine (IL-6, IL-10, IFN-γ, IGF-1, VEGF-C and FGF-2) levels were measured using commercially available ELISA kits (BioLegend, San Diego, CA, USA) according to the manufacturer’s manual and repeated three times. Cytokine levels were normalized by tissue weight and expressed as pg/mg tissue.

### Western blot

After quantification of the protein samples using a Micro BCA™ Protein Assay Kit (Beyotime, Shanghai, China), equivalent amounts of protein (30 µg) were electrophoresed on 12.5% SDS–PAGE gels and then electrotransferred to PVDF membranes (Millipore, Billerica, MA, USA). After blocking, the membranes were incubated at 4 °C overnight with the following primary antibodies: GAPDH (1:1000), EGF-C (1:1000), VEGFR-3 (1:1000), AKT (1:1000), p-AKT (1:1000), ERK1/2 (1:1000), p-ERK1/2 (1:1000), I*κ*Bα (1:1000), p-I*κ*Bα (1:500), NF-*κ*B p65 (1:1000), and p-NF-*κ*B p65 (1:000) and then incubated with the aforementioned anti-mouse or anti-rabbit secondary antibodies (1:2000) at 37 °C for 1 h. Finally, chromogenic results of the target protein were visualized with an Odyssey infrared laser imaging system (Licor, Lincoln, NE, USA). VEGF-C and VEGFR-3 antibodies were purchased from Abcam (Cambridge, UK), and the other antibodies were purchased from CST (Danvers, MA, USA). The intensity of the selected bands was quantified and analysed using ImageJ software (National Institutes of Health, Bethesda, MD, USA).

### 16s rRNA sequencing

Fresh faeces collected in sterile tubes were frozen at − 80 °C for microbiota analysis. In brief, approximately 200 mg of each faecal sample was processed for DNA extraction using the E.Z.N.A.® soil DNA Kit (Omega Bio-tek, Norcross, GA, U.S.) according to the manufacturer’s protocols. The final DNA quality and quantity were assessed using 1% agarose gel electrophoresis and a spectrophotometer. The V3-V4 hypervariable regions of the bacterial 16S rRNA gene were amplified with primers 341F (5′-CCTAYGGGRBGCASCAG-3′) and 806R (5′-GGACTACHVGGGTWT CTAAT-3′) by a thermocycler PCR system (GeneAmp 9700, ABI, USA). The resulting PCR products were detected by 2% agarose electrophoresis using Quantifluor-ST (Promega, USA) and quantified according to the Illumina NovaSeq 6000 platform (Illumina, San Diego, USA). Standard operating procedures for generating equimolar and 2 × 300 bp paired-end sequencing libraries from purified amplified fragments.

### 16S rRNA sequence bioinformatics analysis

The sequences were demultiplexed and quality filtered using QIIME2 docs along with customized program scripts (https://docs.qiime2.org/2021.4/). Briefly, raw data FASTQ files were imported into the format, which could be operated by the QIIME2 system using the tool import program. Demultiplexed sequences from each sample were quality filtered and trimmed, denoised, and merged, and then the chimeric sequences were identified and removed using the DADA2 plugin within QIIME2 to obtain the feature table of amplicon sequence variants (ASVs). The QIIME2 feature-classifier plugin was then used to align ASV sequences to the Silva reference database (V.138.1) to generate the taxonomy. Alpha diversity indices, including the Shannon and Simpson diversity indices, were calculated to estimate microbial diversity within an individual sample. Beta diversity comparisons among groups were visualized via principal coordinate analysis (PCoA) using Bray–Curtis distance. The taxonomy of each 16S rRNA sequence was analysed by the RDP Classifier algorithm against the Silva database, and the community composition was analysed at the genus and phylum levels. Cladogram plot and linear discriminant analysis (LDA) effect size (LEfSe) analyses of 16S rRNA sequences were carried out using Bioincloud software (https://www.bioincloud.tech/) to identify corresponding group biomarkers. The LDA score (log_10_) > 2 was considered to be the criterion with statistical significance.

### Statistics analysis

To ensure the veracity of this study, all experiments were repeated in triplicate, and all data were expressed as the means ± standard deviation (SD) unless otherwise indicated. The general statistical analyses were performed with SPSS version 20.0 (SPSS Inc., Chicago, IL, USA) and Prism version 9.0 (GraphPad Software, La Jolla, CA, USA). Numerical variables between groups were tested by unpaired Student’s *t* test or one-way analysis of variance (ANOVA). Statistical microbiota analyses were performed in QIIME. Comparisons were performed at different taxonomic levels. ANOVA was used to test differences among groups for the diversity indices and relative abundance of the core microbiota followed by Tukey correction or Kruskal–Wallis test. *P* values less than 0.05 were regarded as statistically significant.

## Results

### Isolation, identification and homing of ADSCs

**Fig. 2:**
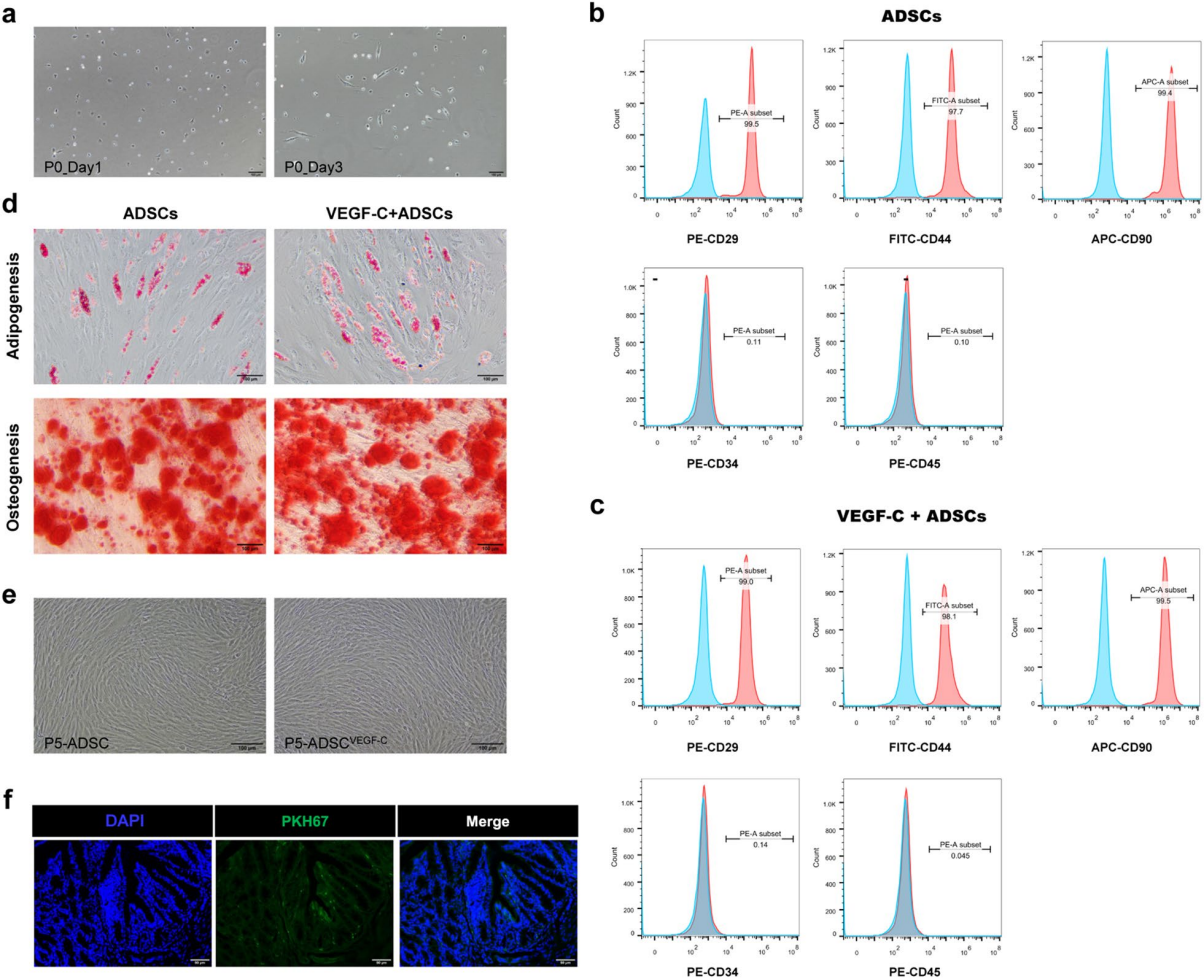
Characterization and identification of isolated ADSCs and VEGF-C stimulated ADSCs in vitro and homing of ADSCs in vivo. **a** Images of ADSCs in the primary passage on Day 1 and Day 3. Scale bar 100 μm; ×100, magnification. **b**, **c** Flow cytometry analysis of surface immunophenotype in the ADSCs and VEGF-C pretreated ADSCs, respectively. The blue line represents blank control and the red line represents marked ADSCs. **d** Detection of the multipotent capacity of ADSCs to induce adipogenic and osteogenic differentiation by Oil Red O and Alizarin Red staining, respectively. Scale bar 100 μm; × 200, magnification. **e** Images of ADSCs unstimulated and stimulated with VEGF-C at the 5th passage. Scale bar 100 μm; × 100, magnification. **f** The distribution of the injected PKH67-labelled ADSCs (green) in the murine colon under a fluorescence microscope with DAPI (blue). Scale bar 100 μm; × 200, magnification

Primary stem cells were isolated from human abdominal subcutaneous adipose tissues and cultured for 10 days. The tiny spindle-shaped morphology of cells was observed on Day 1 and Day 3 (Fig. 2a). At passage 4, flow cytometric analyses confirmed that the isolated ADSCs and VEGF-C stimulated ADSCs both positively expressed the MSC surface markers CD29 (PE; 99.5% vs. 99.0%), CD44 (FITC; 97.7% vs. 98.1%), and CD90 (APC; 99.4% vs. 99.5%) and negatively expressed the endothelial or haematopoietic markers CD34 (PE; 0.11% vs. 0.14%) and CD45 (PE; 0.10% vs. 0.045%) (Fig. 2b, c). To further verify the multilineage differentiation potential of ADSCs, adipogenic differentiation by Oil Red O staining demonstrated that ADSCs formed lipid droplets after 10 days of induction, and osteogenic differentiation by Alizarin Red staining showed that calcium nodules appeared in ADSCs after 14 days of induction (Fig. 2d). Before application in vivo, 100 ng/ml recombinant human VEGF-C protein was cocultured with ADSCs at the fifth passage for 48 h. The morphology and viability of stem cells were not significantly different between ADSCs with and without pretreatment with VEGF-C (99.8% vs. 99.4%, Fig. 2e).

Next, to confirm the homing of ADSCs in the intestine, PKH67-labelled ADSCs were intraperitoneally administered during the second cycle of cell therapy. Five days after injection, PKH67-labelled ADSCs were observed within the colonic mucosa and MLN with green fluorescence, suggesting that ADSCs could home to the inflamed colons and MLN (Fig. 2f and Additional file 2: Fig. S1).

### Paracrine function, effects of secretome on LECs migration and VEGF-C/VEGFR-3 expression in ADSCs and ADSCs stimulated by VEGF-C in vitro

**Fig. 3:**
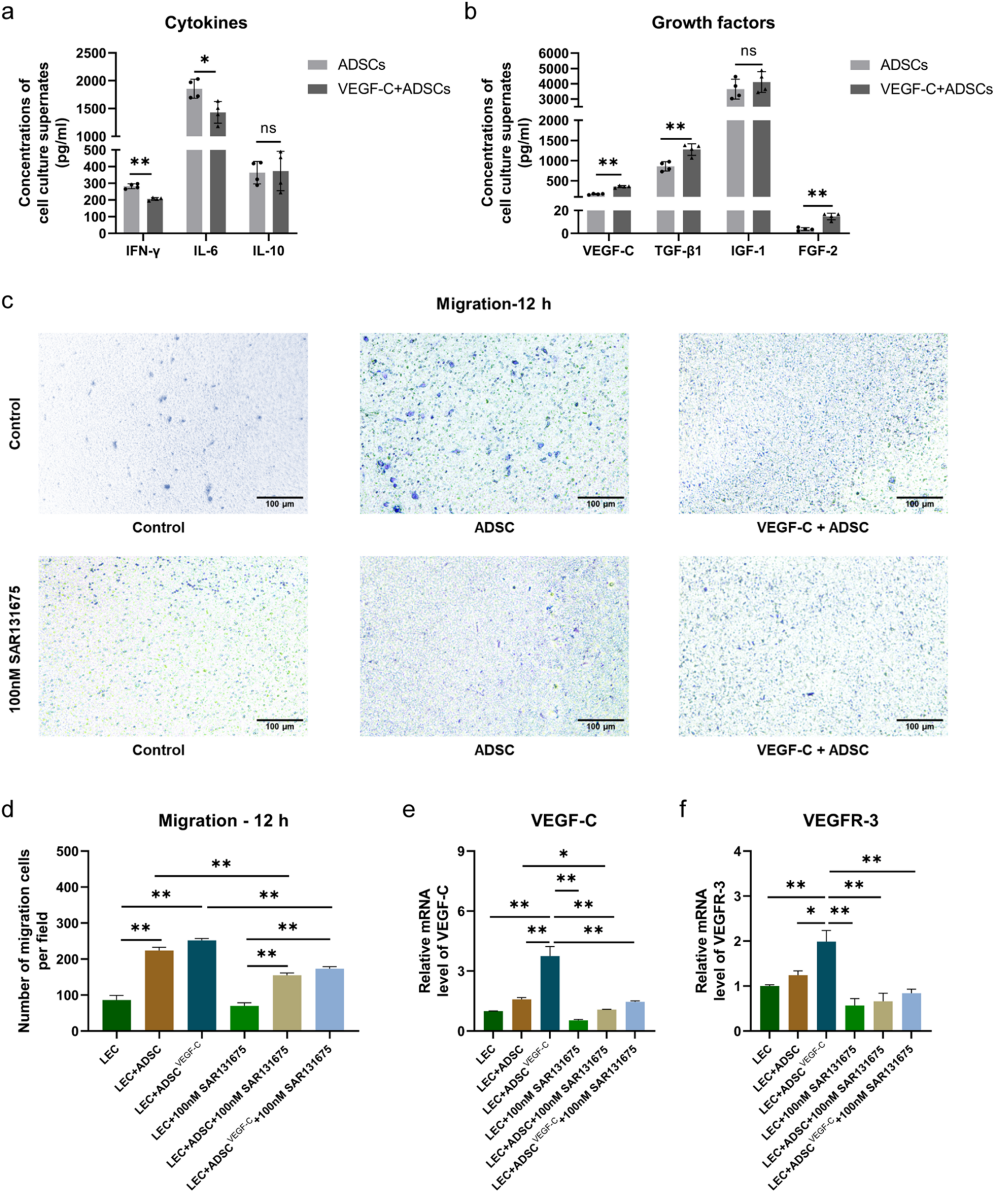
Secretome in the supernatants of ADSCs stimulated with or without VEGF-C in vitro and their effects of LECs migration, VEGF-C/VEGFR-3 expression. **a** Concentrations of proinflammatory cytokines (IFN-γ, IL-6) and anti-inflammatory cytokines (IL-10) in cell culture supernatants were detected by ELISA. **b** Concentrations of growth factors (VEGF-C, TGF-β1, IGF-1, FGF-2) in cell culture supernatants detected by ELISA. (*N* = 4, *P < 0.05, **P < 0.01) **c** Representative images of LEC migration per group were captured at 12 h. Scale bar 100 μm; × 100, magnifications. **d** Quantification of transwell migration assay data. **e** Relative mRNA expression levels of VEGF-C. **f** Relative mRNA expression levels of VEGFR-3. (*N* = 3, *P < 0.05, **P < 0.01). ELISA results are shown as the means ± standard deviation (SD) while migration count and mRNA data are presented as the means ± standard errors (SEM)

Upon reaching approximately 70% confluency at the fifth passage, half of the ADSCs supernatants were stimulated by VEGF-C for 48 h. To avoid the influence of exogenous VEGF-C protein affecting VEGF-C concentration, we detected the concentration of VEGF-C by ELISA in the culture supernatants of ADSCs treated with VEGF-C after being washed and collected both cell supernatants after 24 h of reseeding. Compared with the ADSCs group, ELISA results showed that the levels of the proinflammatory cytokines IL-6 and IFN-γ in the VEGF-C + ADSCs group significantly decreased (IL-6,1855.0 ± 170.6 pg/ml vs. 1431.0 ± 193.7 pg/ml; IFN-γ, 282.3 ± 14.6 pg/ml vs. 206.2 ± 8.4 pg/ml; both *P* < 0.05), respectively; however, no major difference was detected in the anti-inflammatory cytokine IL-10 (363.5 ± 67.7 pg/ml vs. 373.4 ± 118.0 pg/ml; *P* > 0.05, Fig. 3a). Moreover, the concentrations of TGF-β1 and FGF-2 were higher in the VEGF-C + ADSCs than in the ADSCs group (TGF-β1, 1274.0 ± 144.0 pg/ml vs. 857.2 ± 115.3 pg/ml; FGF-2, 14.7 ± 2.9 pg/ml vs. 3.7 ± 1.3 pg/ml; *P* < 0.05, Fig. 3b), respectively. No marked difference was found in the content of IGF-1 between the two groups (4110.0 ± 677.5 pg/ml vs. 3649.0 ± 648.4 pg/ml; *P* > 0.05). The level of VEGF-C in the VEGF-C-stimulated ADSCs had a higher concentration than that in ADSCs group (356.0 ± 25.9 pg/ml vs. 168.8 ± 12.9 pg/ml; *P* < 0.05). In general, VEGF-C stimulation efficiently altered the paracrine function of ADSCs to some extent.

For further observation the effects of ADSCs secretome on LECs, we performed transwell migration assay in vitro. Cultivating ADSCs and VEGF-C stimulated ADSCs in the lower chamber significantly enhanced LEC migration ability compared to the control group (224.00 ± 8.74 vs. 86.33 ± 12.44, 251.70 ± 5.78, 86.33 ± 12.44; all *P* > 0.05, Fig. 3c, d). Meanwhile, 100 nM VEGFR-3 inhibitor (SAR131675) treated LECs per group showed attenuated transmigration. Compared with LEC and ADSCs cocultured LECs, VEGF-C treatment obviously increased both VEGF-C and VEGFR-3 mRNA expression (all *P* < 0.05, Fig. 3e, f). After VEGFR-3 inhibitor were added in the coculture system, VEGF-C pretreatment could promote the expression of VEGF-C and VEGFR-3, but there were no statistical differences.

### VEGF-C-stimulated ADSCs alleviated intestinal inflammation and disease progression in chronic experimental colitis

**Fig. 4:**
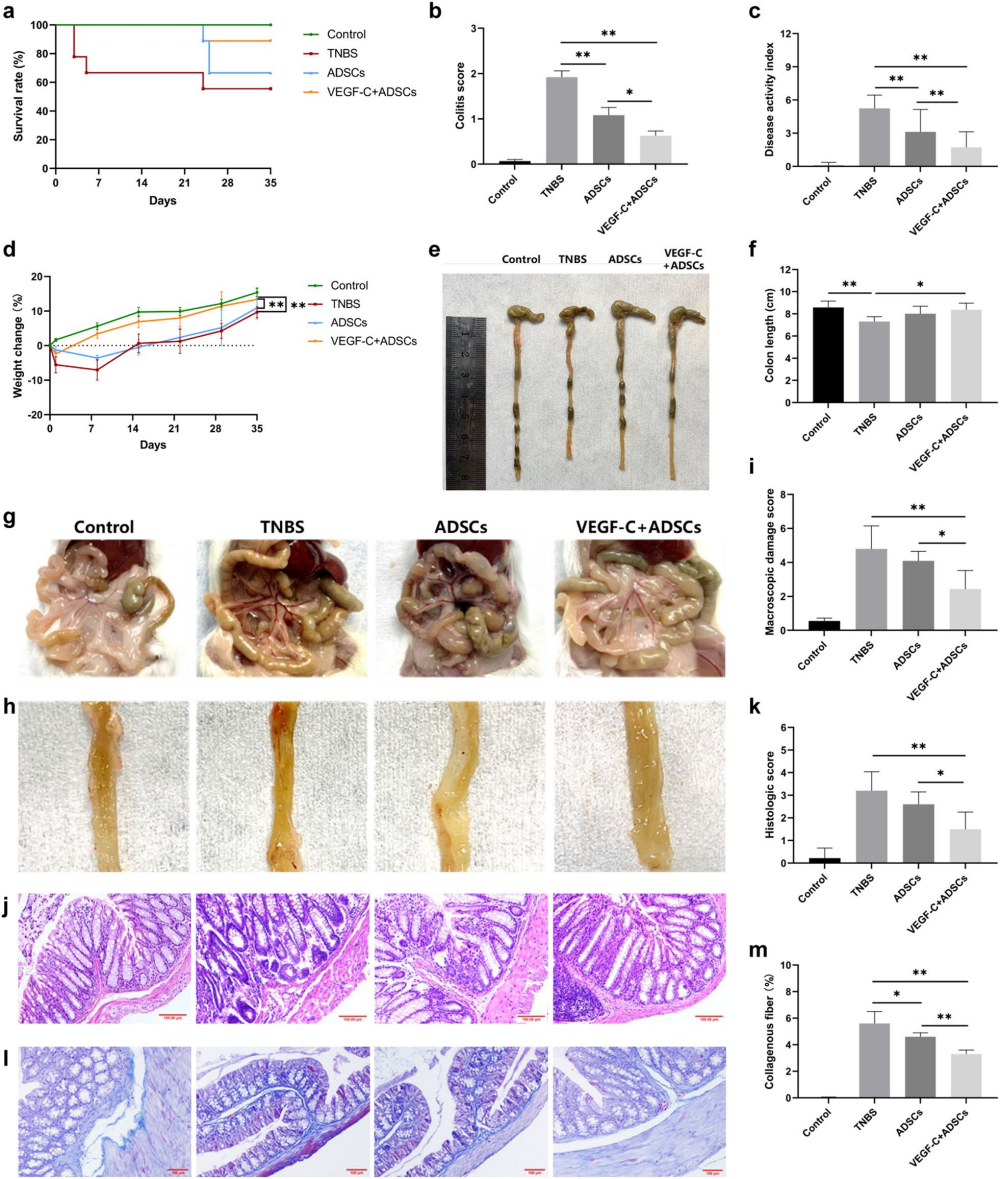
The therapeutic effects of VEGF-C-stimulated ADSCs on TNBS-induced colitis. **a** Survival rate. *N* = 9 mice per group. **b** Colitis score. **c** DAI based on body weight changes, stool traits, and haematochezia. **d** Weight changes. **e** Representative images of colon length per group. **f** Comparison of colon length. **g** Representative images of colon macroscopic damage per group. **h** Representative images of mucosal lesions per group. **i** Macroscopic damage score. **j** Representative images of representative H&E staining images per group. Scale bar 100 μm; ×200, magnifications. **k** Histological score. **l** Representative Masson’s trichrome-stained image per group. Scale bar 100 μm; ×200, magnifications. **m** Collagen deposition was quantified as a ratio of the positive area to the total area of colon tissues. (*N* ≥ 5, *P < 0.05, **P < 0.01)

To evaluate whether VEGF-C-stimulated ADSCs are more potent than ADSCs treatment alone, intestinal inflammation was detected in the mice with TNBS-induced chronic colitis. From the first TNBS induction, there was no significant difference in overall survival rate seen between the two treatment groups, although the mice had prolonged survival time in the VEGF-C + ADSCs group (*P* > 0.05, Fig. 4a). Compared with the TNBS group, the colitis score was significantly reduced in both the ADSCs group (1.08 ± 0.86 vs. 1.92 ± 0.70; *P* < 0.01) and VEGF-C + ADSCs group (0.63 ± 0.67 vs. 1.92 ± 0.70; *P* < 0.01, Fig. 4b). Coinciding with the colitis score, the results were similar in DAI (ADSCs vs. TNBS: 3.12 ± 2.03 vs. 5.24 ± 1.20; VEGF-C + ADSCs vs. TNBS: 1.73 ± 1.41 vs. 5.24 ± 1.20; both *P* < 0.01, Fig. 4c). Meanwhile, these scores in the VEGF-C + ADSCs group were significantly lower than those in the ADSCs group (*P* < 0.05, Fig. 4b, c). Additionally, body weight in the VEGF-C + ADSCs group increased more rapidly than that in the ADSCs group (13.39 ± 9.50% vs. 10.95 ± 7.94%; *P* < 0.01) and the TNBS group (13.39 ± 9.50% vs. 9.06 ± 3.28%; *P* < 0.01, Fig. 4d, Additional file 1: Table 6).

After being sacrificed, the mice in the TNBS group presented mesenteric hyperaemia, local intestinal strictures, and adhesion between the intestine and adjacent abdominal tissues in the abdominal cavity (Fig. 4g). The colon mucosa also displayed congestion, oedema and superficial ulcer formation (Fig. 4h). However, these manifestations were alleviated after ADSCs or VEGF-C + ADSCs treatment. The colon length in the VEGF-C + ADSCs group was obviously longer than that in the TNBS group (8.36 ± 0.60 cm vs. 7.30 ± 0.45 cm; *P* < 0.05), but there was no statistical difference compared with that in the ADSCs group (*P* > 0.05, Fig. 4e, f). The macroscopic damage score in the mice administered VEGF-C stimulated ADSCs was obviously lower than that in the TNBS group (2.44 ± 1.08 vs. 4.80 ± 1.35; *P* < 0.01) and ADSCs group (2.44 ± 1.08 vs. 4.10 ± 0.55; *P* < 0.05, Fig. 4i).

Furthermore, histological changes in TNBS group mice showed increased numbers of inflammatory cells infiltration in colonic tissues with disorganized crypt architecture, crypt abscesses and decreased goblet cells compared with the two treatment groups (Fig. 4j). Microscopically, colonic inflammation in the VEGF-C + ADSCs group was markedly alleviated compared with that in the TNBS (1.50 ± 0.76 vs. 3.20 ± 0.84; *P* < 0.01) and ADSCs groups (1.50 ± 0.76 vs. 2.60 ± 0.55; *P* < 0.05, Fig. 4k). Additionally, submucosal fibrosis was assessed by Masson’s trichrome staining. Collagenous fibre hyperplasia was reduced in the VEGF-C + ADSCs group (Fig. 4l). Quantification of the staining-positive area was prominently smaller in the VEGF-C + ADSCs group than in the TNBS (3.30 ± 0.30% vs. 4.60 ± 0.31%; *P* < 0.01) and ADSCs groups (3.30 ± 0.30% vs. 5.61 ± 0.90%; *P* < 0.01, Fig. 4m).

### ADSCs promoted lymphangiogenesis and enhanced lymphatic drainage after stimulation with VEGF-C

**Fig. 5:**
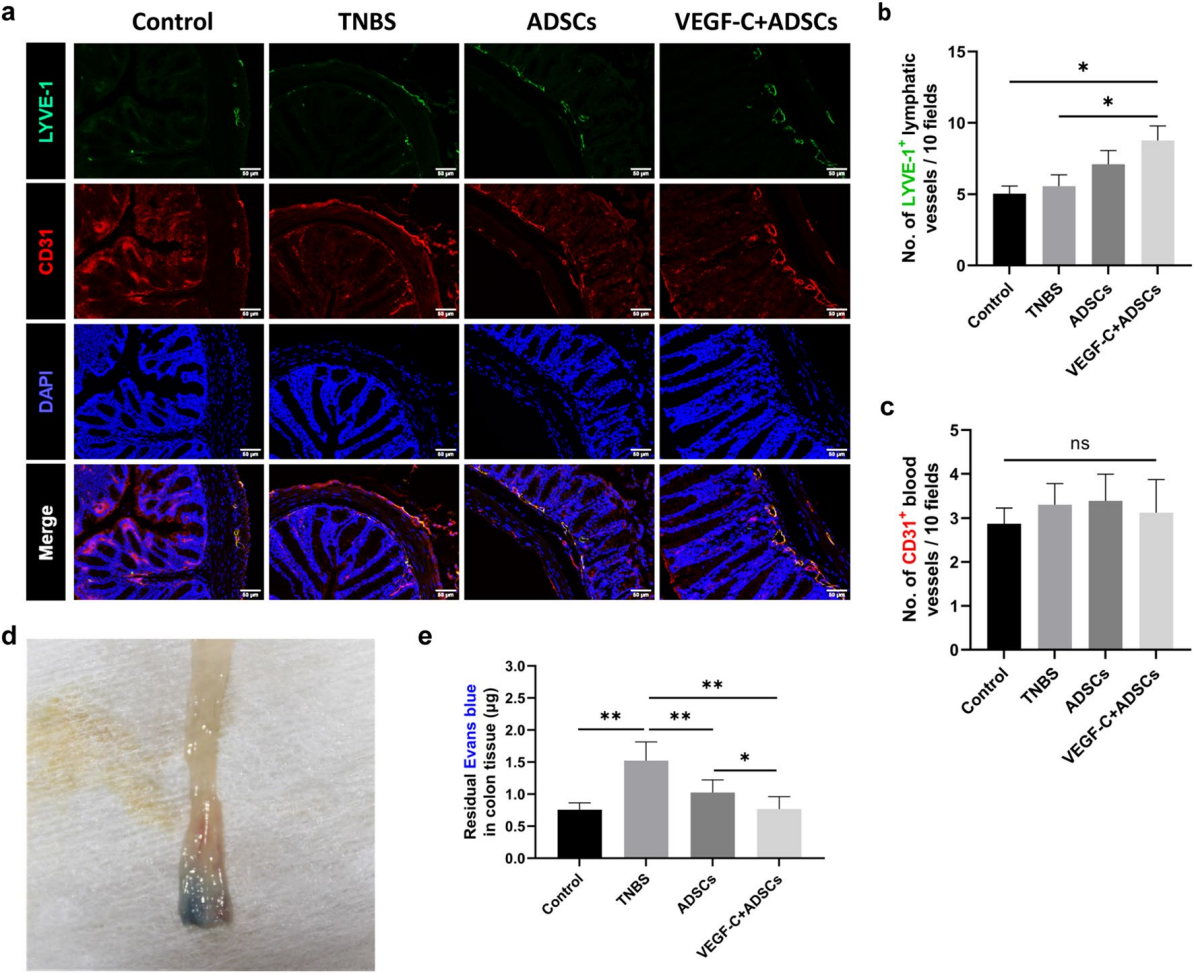
Evaluating colonic formation of lymphatic vessels and lymphatic drainage. **a** Immunofluorescence staining of LYVE-1^+^ lymphatic vessels and CD31.^+^ blood vessels. Scale bar 50 μm; ×200, magnification. **b** Lymphatic vessel density. **c** Microvascular density. **d** Residual Evans blue dye of the terminal colon. **e** Analysis of lymphatic drainage with rectal submucosal injection of Evans blue dye. (*N* ≥ 4, *P < 0.05, **P < 0.01)

The lymphatic vessels (LYVE-1^+^ staining) and blood vessels (CD31^+^ labelling) were detected by immunofluorescence assay, and positive microvascular structures were mainly located at the submucosal layer (Fig. 5a). Chronic colitis mice treated with ADSCs stimulated with VEGF-C substantially increased the LVD in comparison to TNBS-treated mice (8.77 ± 3.20 vs. 5.55 ± 2.55; *P* < 0.05) and the control mice (8.77 ± 3.20 vs. 5.05 ± 1.67; *P* < 0.05, Fig. 5b). However, no apparent differences in LVD and MVD were found between the VEGF-C + ADSCs and ADSCs groups (8.77 ± 3.20 vs. 7.10 ± 3.00, 3.13 ± 2.36 vs. 3.39 ± 1.93; *P* > 0.05, Fig. 5c).

To investigate whether ADSCs pretreatment with VEGF-C could improve lymphatic drainage, Evans blue dye was injected into the rectal submucosa of mice (Fig. 5d). The residual Evans blue dye in the VEGF-C + ADSCs group was expressively diminished compared with that in the ADSCs group (0.77 ± 0.20 μg vs. 1.03 ± 0.20 μg; *P* < 0.05) and TNBS group (0.77 ± 0.20 μg vs. 1.52 ± 0.29 μg; *P* < 0.05). Taken together, lymphatic drainage was significantly enhanced in the VEGF-C + ADSCs group compared to the ADSCs group (Fig. 5e).

### VEGF-C-stimulated ADSCs improved pro- and anti-inflammatory balance by increasing the secretome

**Fig. 6:**
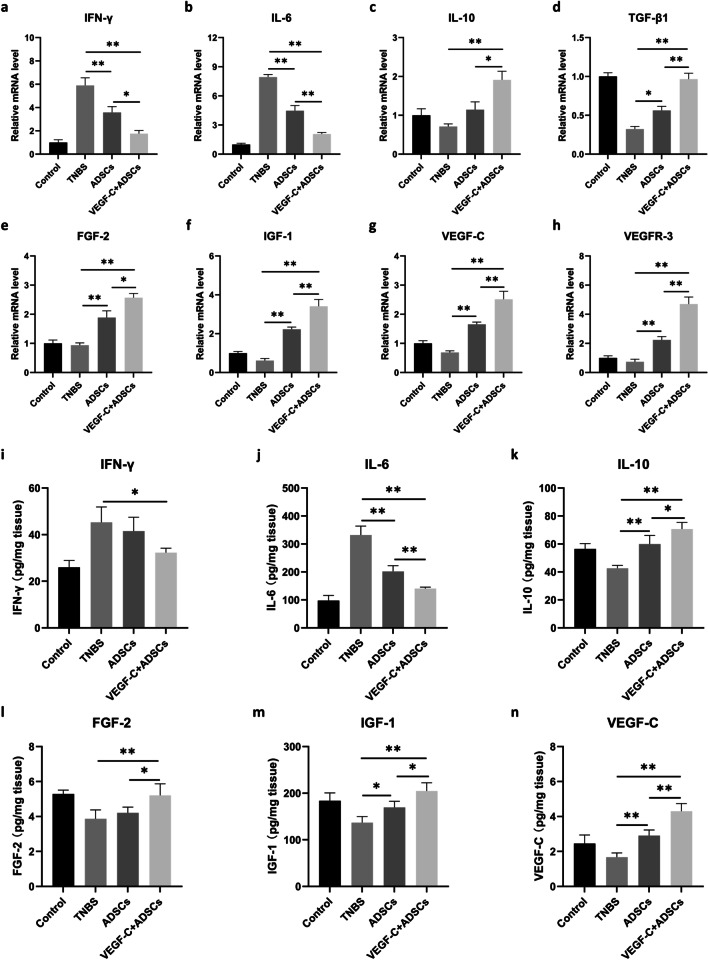
VEGF-C pretreatment helped build up pro- and anti-inflammatory balance via the increment of growth factors. The relative mRNA expression levels of **a** IFN-γ, **b** IL-6, **c** IL-10, **d** TGF-β1, **e** FGF-2, **f** IGF-1, **g** VEGF-C and **h** VEGFR-3 in the colons. The protein concentrations of **i** IFN-γ, **j** IL-6, **k** IL-10, **l** FGF-2, **m** IGF-1 and **n** VEGF-C. mRNA data are presented as the means ± standard errors (SEM) while ELISA results are shown as the means ± standard deviation (SD) (*N* = 4, *P < 0.05, **P < 0.01)

We further analysed the murine colonic mRNA expression of cytokines and growth factors. Compared with the TNBS group and ADSCs group, the mice in the VEGF-C + ADSCs group had strikingly decreased mRNA levels of proinflammatory cytokines, including IFN-γ, TNF-α, IL-1β, IL-6 and IL-17A (*P* < 0.05, Fig. 6a, b and Additional file 3: Fig. S2a–c), as well as increased expression of the anti-inflammatory cytokine IL-10 (*P* < 0.05, Fig. 6c). In line with the in vitro results, the relative mRNA levels of the paracrine growth factors TGF-β1, FGF-2, IGF-1, Ang-2, and VEGF-C produced by stem cells in the VEGF-C + ADSCs group were significantly higher than those in the ADSCs group (*P* < 0.05, Fig. 6d–g and Additional file 3: Fig. S2d). VEGFR-3, the receptor binding to VEGF-C, also had markedly elevated mRNA expression in the VEGF-C + ADSCs group (*P* < 0.05, Fig. 6h).

Additionally, protein levels of the indicated cytokines were determined by ELISA. No significant difference was observed in the expression of proinflammatory cytokine IFN-γ between two ADSCs groups (*P* > 0.05, Fig. 6i). However, compared with the TNBS group and ADSCs group, treatment with VEGF-C-stimulated ADSCs inhibited the elevation of IL-6 (*P* < 0.05, Fig. 6j). Similar to the mRNA levels, the protein concentrations of IL-10, FGF-2, IGF-1, and VEGF-C in colons were the highest in VEGF-C + ADSCs group (*P* < 0.05, Fig. 6k-n).

Collectively, these data suggested that ADSCs stimulated by VEGF-C had the ability to modulate colonic mucosal pro- and anti-inflammatory dynamic changes by promsoting secretome-composing cytokines and paracrine growth factors.

### ADSCs stimulated by VEGF-C alleviated intestinal inflammation via upregulation of the VEGF-C/VEGFR-3 axis-mediated mechanism and inhibition of the NF-κB signalling pathway

**Fig. 7:**
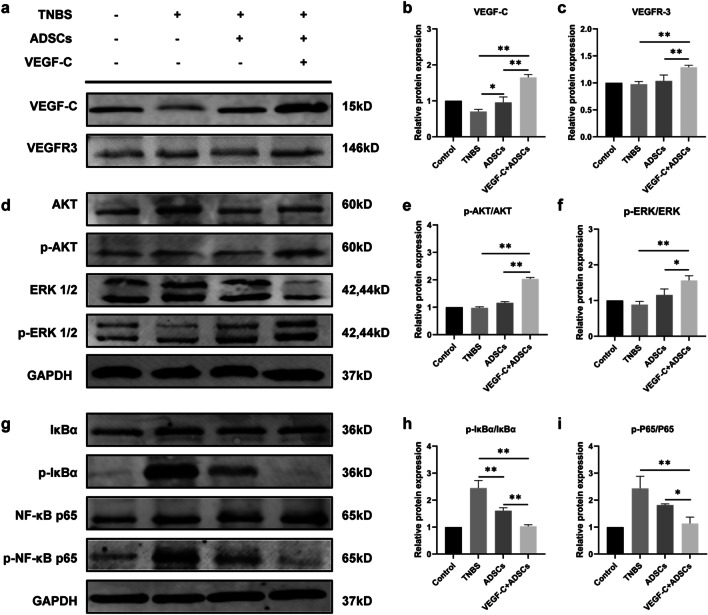
VEGF-C-stimulated ADSCs activated a VEGF-C/VEGFR-3-dependent mechanism and suppressed the NF-*κ*B pathway in chronic experimental colitis. **a** The protein bands of VEGF-C and VEGFR-3. **b** Quantification of VEGF-C relative to GAPDH. **c** Quantification of VEGFR-3 relative to GAPDH. **d** The protein bands of AKT, p-AKT, ERK1/2 and p-ERK1/2. **e** Quantification of p-AKT relative to AKT. **f** Quantification of p-ERK1/2 relative to ERK1/2. **g** The protein bands of I*κ*Bα, p-I*κ*Bα, NF-*κ*B p65 and p-NF-*κ*B p65. **h** Quantification of p-I*κ*Bα relative to I*κ*Bα. **i** Quantification of p-NF-*κ*B p65 relative to NF-*κ*B p65. (*N* = 4, *P < 0.05, **P < 0.01)

To investigate the mechanisms of ADSCs stimulated by VEGF-C in the treatment of chronic colitis, we detected the possible contribution of VEGF-C/VEGFR-3 signalling, which is the key molecular mechanism in LEC proliferation and lymphangiogenesis. Western blotting analysis verified that both VEGF-C and VEGFR-3 protein were highly expressed in the murine colons of the VEGF-C + ADSCs group compared with the TNBS and ADSCs groups (*P* < 0.05, Fig. 7a-c). Accordingly, two VEGFR-3-mediated downstream signalling pathways, the AKT and ERK pathways, were also studied. The protein levels of phosphorylated AKT (p-AKT) and phosphorylated ERK1/2 (p-ERK1/2) were significantly upregulated in the mice treated with VEGF-C + ADSCs compared with those in the ADSCs and TNBS groups (*P* < 0.05, Fig. 7d-f). These results indicated that VEGF-C-pretreated ADSCs could upregulate the VEGF-C/VEGFR-3 pathway, activate the downstream AKT and ERK pathways within intestinal LECs, and eventually induce the formation of submucosal lymphatic vessels and improve lymphatic drainage.

Subsequently, we further explored the mechanism relevant to colonic inflammation. Our studies revealed that p-I*κ*Bα and NF-*κ*B p65 were significantly downregulated in the VEGF-C + ADSCs group compared with the TNBS and ADSCs groups (*P* < 0.05, Fig. 7g-i). No noticeable differences were found in total I*κ*Bα and NF-*κ*B p65 protein levels among all groups (*P* > 0.05, Fig. 7g). Thus, VEGF-C-stimulated ADSCs were also able to effectively inhibit the NF-*κ*B signalling pathway to relieve intestinal inflammation.

### VEGF-C-stimulated ADSCs significantly increased lymphatic drainage of colonial immune cells to MLNs

**Fig. 8:**
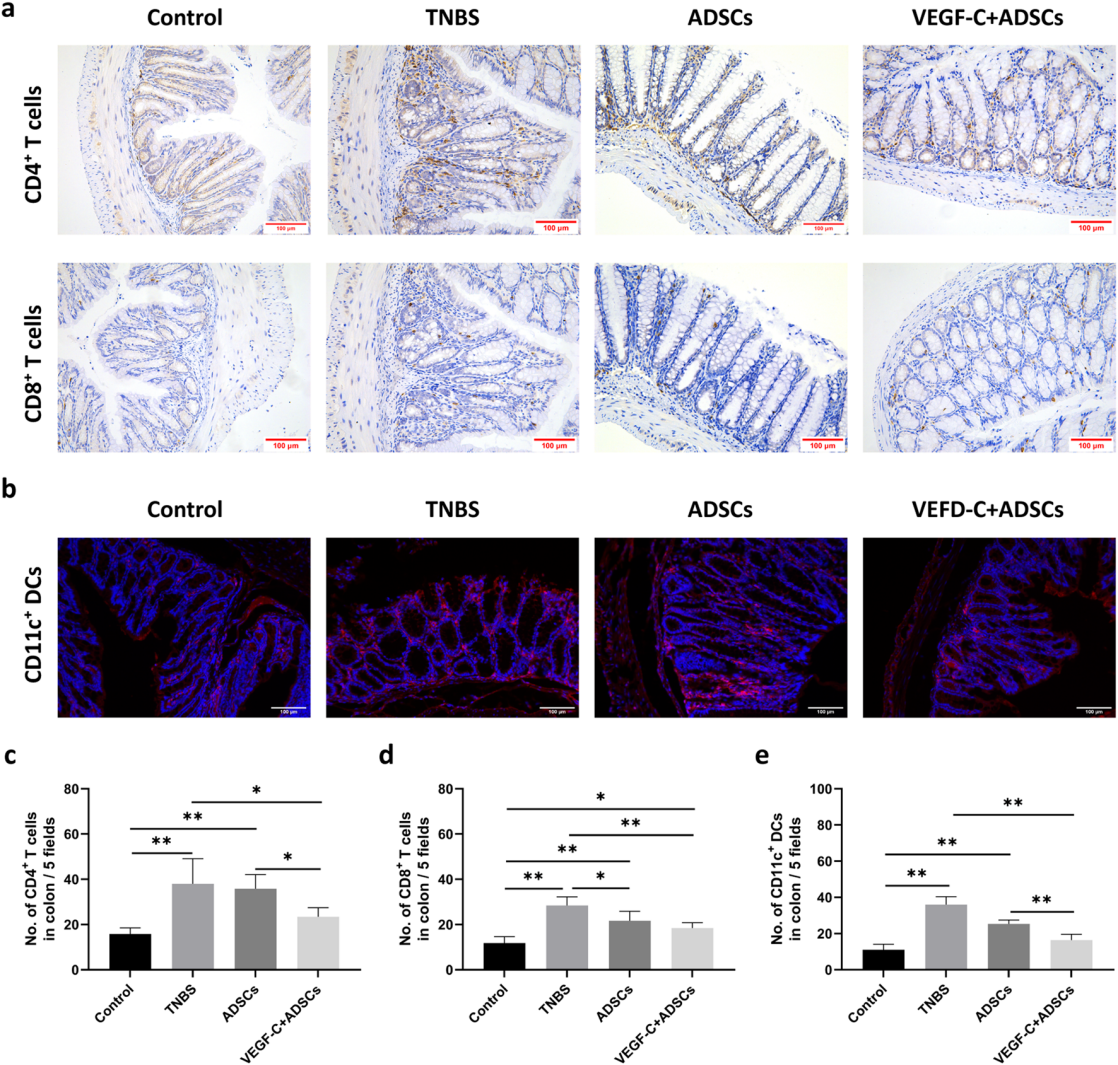
Immunomodulatory functions of ADSCs and VEGF-C + ADSCs on immune cells in the colon and MLNs. **a** CD4^+^ T cells and CD8^+^ T cells in the colon tissues were observed by IHC. ×200, magnification. **b** CD11c^+^ dendritic cells in the colon tissues were observed by IF. ×200, magnification. **c** Colonic CD4^+^ T cells. **d** Colonic CD8^+^ T cells. **e** Colonic CD11c^+^ dendritic cells. All immune cells were counted per field (*N* = 5) using ImageJ. (*N* = 3, *P < 0.05, **P < 0.01)

Next, we evaluated the effects of enhanced lymphatic drainage function on draining immune cells from inflamed colons to local MLNs. After TNBS attacks, numerous T lymphocytes and CD11c^+^ DCs are concentrated in the colonic mucosal epithelium and lamina propria. Staining results revealed that the numbers of CD4^+^ T cells, CD8^+^ T cells and CD11c^+^ DCs in the colon of the TNBS group were increased compared with those in the two treatment groups (Fig. 8a, b). Compared with the ADSCs group, the numbers of colonic CD4^+^ T cells and CD11c^+^ DCs were remarkably decreased in the VEGF-C + ADSCs group (23.40 ± 4.04 vs. 35.80 ± 6.30; 16.40 ± 3.21 vs. 25.40 ± 2.08, *P* < 0.05; Fig. 8c, e). However, no obvious difference was found in colonic CD8^+^ T cells (*P* > 0.05, Fig. 8d). In the MLN sections, there were no significant differences in the numbers of T cells between the VEGF-C + ADSCs group and the ADSCs group (*P* > 0.05, Additional file 4: Fig. S3a-c). We also found that spleens of the TNBS group had the highest number of both CD4^+^ and CD8^+^ T cells, but no apparent differences could be found between the two treatment groups (Additional file 5: Fig. S4a–c).

### ADSCs stimulated by VEGF-C altered faecal microbiota compositions

**Fig. 9:**
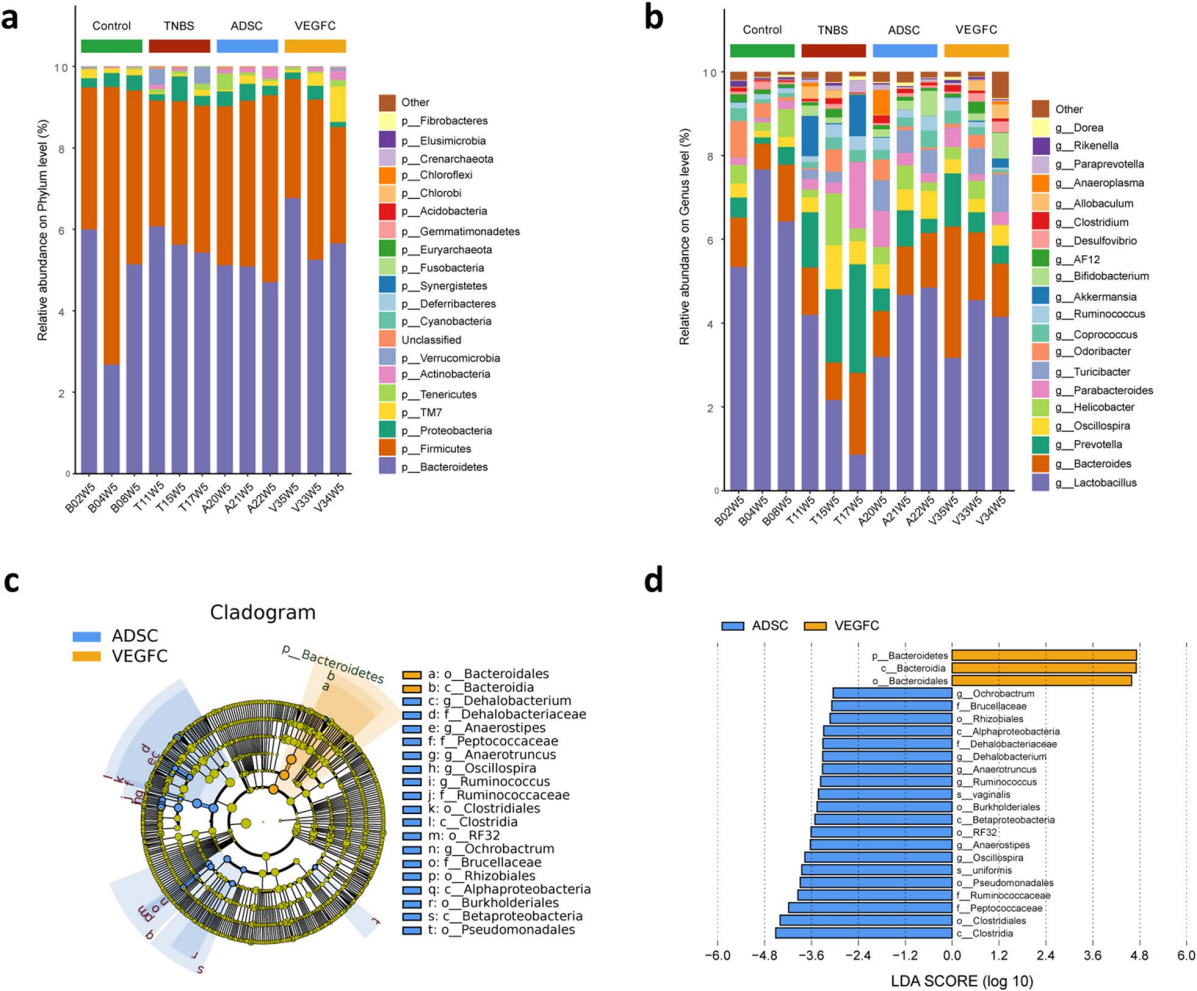
Diversity and abundance analysis of the faecal microbiota of TNBS-induced colitis mice after treatment. **a** Taxonomic profiling revealed differences at the phylum level. Each column displayed an individual mouse and the top 20 most abundant bacteria. **b** Taxonomic profiling revealed differences at the genus level. Each column displayed an individual mouse and the top 20 most abundant bacteria. **c** Taxonomic cladogram obtained from LEfSe analysis. **d** Linear discriminant analysis (LDA) score for different taxa abundances. (*N* = 3, *P < 0.05, **P < 0.01)

As changes in the intestinal flora are usually connected with inflammation status and drainage function of lymphatic vessels, we also investigated the faecal microbiota dynamics related to ADSCs treatment. There were no distinct variations in alpha and beta diversity among the four groups (*P* > 0.05, Additional file 6: Fig. S5a–c). At the phylum level, compared with the ADSCs group, the relative abundance of *p-Firmicutes* was increased and *p-Bacteroidetes* was decreased in the TNBS group and the VEGF-C + ADSCs group (*P* < 0.05, Fig. 9a and Additional file 6: Fig. S5d). At the genus level, in contrast to the microbiota in the TNBS group, the higher abundance of *g-Lactobacillus* and lower *g-Prevotella* were strikingly observed in the ADSCs and VEGF-C + ADSCs groups (*P* < 0.05, Fig. 9b and Additional file 6: Fig. S5e).

In addition, to identify discriminating features between two treatment groups, cladograms and LEfSe analysis were applied to identify the key microbial markers. Ten remarkable differences were revealed in the ADSCs group, and three significant microbial features were observed in the VEGF-C + ADSCs group (Fig. 9c, d). After VEGF-C-stimulated ADSCs treatment, we found that *p-Bacteroidetes*, *c-Bacteroidia* and *o-Bacteroidales* were all noticeably enriched in faeces. In the mice injected with ADSCs alone, the generic abundances of *Oscillospira*, *Ruminococcus*, *Anaerotruncus*, *Ochrobactrum*, *Dehalobacterium* and *Anaerostipes* exhibited a significant decrease. Among these, *Ochrobactrum* belongs to the *Proteobacteria*, while the other five genera belong to the *Firmicutes*.

## Discussion

**Fig. 10:**
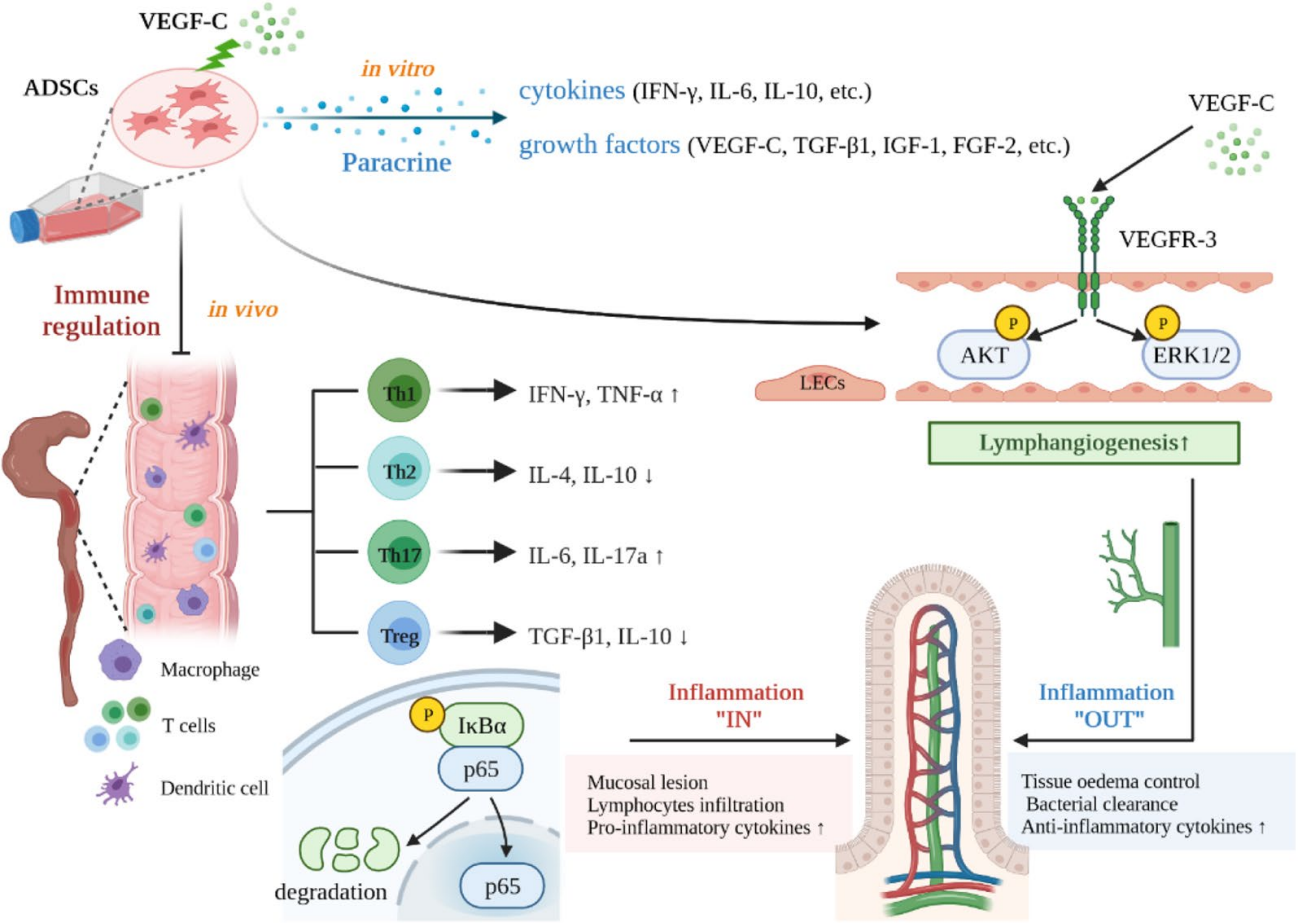
Schematic depiction of the underlying mechanisms by which VEGF-C-stimulated ADSCs alleviate chronic colitis via lymphangiogenesis and immunomodulatory effects. VEGF-C-stimulated ADSCs could simultaneously improve lymphatic drainage (“OUT”) and inhibit the inflammatory response (“IN”) by activating VEGF-C/VEGFR-3-dependent signals and suppressing the NF-*κ*B pathway

ADSCs have been found to exhibit anti-inflammatory and immunomodulatory properties that report high expectations in various fields. In this study, we investigated the therapeutic effects of VEGF-C-stimulated ADSCs on CD and the underlying mechanisms. Our results demonstrated that: (1) secretion of ADSCs altered after VEGF-C coculture, but immunophenotype, differentiation potential and homing ability of ADCSs did not change; (2) administration of VEGF-C-stimulated ADSCs markedly alleviated colonic pathological lesions, and ameliorated lymphatic drainage to enhance inflammation-associated immune cells removing from the inflamed gut; (3) VEGF-C pretreatment improved pro- and anti-inflammatory balance; (4) the compositions of faecal microbiota changed after treatment; (5) activation of VEGF-C/VEGFR-3 dependent mechanism and inhibition of NF-*κ*B signalling pathway regulated this process (Fig. 10).

In previous studies, researchers paid little attention to the role of lymphatics in the pathophysiology of CD. The intestinal and mesenteric lymphatic vessels conduct functional and morphological changes in CD patients, which negatively influence intestinal immune homeostasis, stable faecal microbiota, and epithelial barrier integrity [22]. VEGF-C, as the main ligand of VEGFR-3, is necessary for regulating functional lymphatic vessel networks [36]. Our previous results confirmed that VEGF-C treatment enhanced intestinal lymphatic drainage in DSS-induced chronic colitis, thereby improving the gut microbiota [27]. Additionally, in acute and chronic colitis, blocking VEGFR-3 exacerbates colitis, impairing lymphatic drainage and structural changes [34]. Based on these studies, we speculated that therapeutic intervention by regulating the key signalling pathways in promoting lymphangiogenesis and lymphatic drainage could improve chronic colitis.

ADSCs transplantation has presented long-term safety and efficacy with low recurrence in refractory perianal fistulizing CD [37, 38]. However, current evidence for systemic administration of MSCs in luminal CD has demonstrated ambiguous efficacy and uncertain mechanisms [6, 39–42]. Therefore, further research is needed to explore the optimization of MSCs therapy. Some findings suggested that preconditioning MSCs in vitro could alter secretory components involved in immunomodulation and paracrine mechanisms, further reinforcing therapeutic effects [10]. One study reported that MSCs pretreated with Ang-2 improved the therapeutic efficacy of myocardial infarction through enhancing paracrine regulation (higher production of VEGF and von Willebrand factor) and angiogenesis [43]. Another study showed that after ADSCs were stimulated short-term by VEGF-C, pro-lymphangiogenic growth factors (VEGF-A, VEGF-C, Prox-1) obviously increased in vitro*,* and more distinct lymphangiogenesis was visible after implantation in vivo, the process of which was also strengthened via blockade of TGF-β1 [16]. In our study, higher levels of growth factors (VEGF-C, TGF-β1 and FGF-2) and lower expression of cytokines (IFN-γ and IL-6) were observed in vivo after pretreatment with VEGF-C. Increased expression of FGF-2 and VEGF-C has been reported to be involved in lymphatic metastasis, while FGF-2 exerted less potent lymphangiogenic capacity than VEGF-C. Moreover, dysregulated microbiota-triggered TGF-β1 influenced FGF-2 production in Treg cells during colitis, and cooperative effects between FGF-2 and IL-17A contributed to the regeneration of damaged intestinal epithelium [44]. Accordingly, we proposed an innovative hypothesis that VEGF-C-stimulated ADSCs could play a dual role by inhibiting inflammation and promoting lymphatic drainage to mitigate colitis.‬‬‬‬‬‬‬‬‬‬‬‬‬‬‬‬‬‬‬‬‬‬‬‬‬‬‬‬‬‬

Chronic transmural colitis was induced with escalating doses of TNBS, closely resembling histopathological characteristics in human CD [30, 33]. Obvious inflammatory cell infiltration usually occurs 2 h after TNBS administration, so we executed injection of cells within 2 h. Some researchers have found that in the inflammatory microenvironment, injecting exogenous MSCs in vivo has the characteristic of dominant distribution to sites of inflammation without clear mechanism of this effect. Through systemic injection of MSCs in the IBD model, MSCs not only home to the colon, but also home to the spleen, lung and MLN [42, 45, 46]. When we terminated cell therapy, we confirmed that PKH67-labelled ADSCs could home to the inflamed colon and MLNs. This homing activity helped to reduce the mortality of mice and alleviate colitis. Furthermore, our results demonstrated that VEGF-C-stimulated ADSCs ameliorated lymphatic drainage, which might help facilitate the clearance of inflammatory mediators. During the development of CD, pro- and anti-inflammatory immune cytokines/cells are critical as immunoregulatory mediators. TNBS-induced colitis mainly elicits Th1- and Th17-mediated immune responses, along with large amounts of proinflammatory cytokines and chemokines [47–49]. Prior evidence has documented that ADSCs administration can suppress inflammation by inhibiting Th1 and Th17 responses and inducing Treg expression [42, 50]. In these studies, ADSCs treatment markedly depressed the elevation of IFN-γ, IL-1β and IL-6 but enhanced IL-10 levels. Likewise, after ADSCs and VEGF-C + ADSCs treatment, mRNA levels of a similar spectrum of pro- and anti-inflammatory cytokines were detected in our study. Apart from VEGF-C, other growth factors, including angiopoietins, FGFs, IGFs, and inflammatory cytokines are also implicated in lymphatic vessel formation [51]. However, TGF-β1 is known as a negative regulator of LEC proliferation and lymphangiogenesis [52]. The underlying mechanisms for these various cytokines remain unclear. ADSCs can autocrine VEGF-C, promote LEC proliferation and form lymphatics via the VEGF-C/VEGFR-3 axis [53]. After coculture with VEGF-C, ADSCs produced higher levels of VEGF-C, VEGFR-3 and other growth factors (TGF-β1, FGF-2, IGF-1 and Ang-2) through paracrine mechanisms, which was consistent with their expression in vitro. Previous study showed ADSCs secretome strongly enhanced LEC proliferation, tube formation, and migration in vitro, VEGFR-3 inhibitor (SAR131675) could potently inhibit VEGF-C induced LECs migration, which were similar to our results.

ADSCs can also inhibit the proliferation of T lymphocytes and reduce the maturation of monocyte-derived DCs to exert powerful immunosuppressive functions [54, 55]. A study used flow cytometry to monitor changes in immune cells in patients with severe osteoarthritis after locally injecting ADSCs. ADSCs not only release paracrine factors and cytokines to drive local immediate responses but also generate long-term systemic immune homeostasis [56]. CD11c is highly expressed in myeloid and monocyte-derived DCs and provides an effective target for both CD4^+^ and CD8^+^ T cell responses in vivo [57]. Local lymphocyte infiltration is central to the pathophysiology of CD. To study the role of VEGF-C + ADSCs in the long-term immune response, we found that the counts of CD4^+^ and CD8^+^ T cells in the colons, MLNs and spleens were decreased after injecting VEGF-C-stimulated ADSCs compared with untreated to varying degrees. These results might contribute to proving that normal lymphatic structure and enhanced drainage ability can promote the translocation of these inflammatory cells to MLNs.

To date, the crosstalk of ADSCs, immunomodulation, lymphatic alterations and intestinal inflammation are related to several signalling pathways, and their interactive specific mechanisms remain unclarified. The VEGF-C/VEGFR-3 pathway participates in the core signalling axis involved in regulating lymphangiogenesis and inducing the survival, proliferation and migration of LECs. VEGF-C-mediated activation of VEGFR-3 leads to triggering multiple downstream mediators, including AKT and ERK1/2 [23, 24, 58]. C-reactive protein could improve the proangiogenic capability of ADSCs by activating HIF-1α through the CD64/PI3K/AKT and MAPK/ERK pathways [58]. In a mouse model of heart failure, blocking VEGF-C/VEGFR-3-mediated signals (AKT/ERK1/2, calcineurin A/NFATc1/FOXc2 and CX43) resulted in decreased cardiac lymphangiogenesis and increased cardiac dysfunction [59]. Our study showed ADSCs stimulated by exogenous VEGF-C upregulated VEGFR-3 and further enhanced p-AKT and p-ERK1/2 expression. Except for the intestinal lymphatics correlative signalling pathways, we also focused on the mechanism by which ADSCs inhibit inflammation and regulate immunity. NF-*κ*B is markedly activated in CD patients, which promotes the expression of numerous proinflammatory mediators and affects intestinal mucosal inflammation. The immunomodulatory effects of ADSCs on T cell subsets can be achieved in part by inhibiting the activation of NF-*κ*B through the PD-L1/PD-1 and Gal-9/TIM-3 pathways [54]. Additionally, the VEGF-C/VEGFR-3 pathway inhibits TLR4/NF-*κ*B signalling and targets both lymphatic vessels and immune cells as a clinical application for protecting against endotoxin shock [60]. Our results indicated that VEGF-C-stimulated ADSCs treatment prevented the degradation and phosphorylation of I*κ*Bα, leading to reduced release and nuclear translocation of p65 in colon tissues. However, whether the other pathways such as Wnt/β-catenin and Notch signalling pathways are also involved in therapeutic intervention awaits further investigation.

Bacterial clearance is another important function of the intestinal lymphatic system. Interactions between gastrointestinal microbes and ADSCs could enhance the therapeutic effects of CD by inducing immunomodulation and migration of ADSCs [61, 62]. The gut microbiota in CD is characterized by decreased bacterial diversity and imbalanced bacterial composition. However, we did not find distinct differences in the alpha and beta diversity of faecal microbes among all groups in our study. Further, we observed that the microbiome alterations caused by TNBS induction were changed to some extent reversed by the administration of ADSCs and VEGF-C-stimulated ADSCs. The analysis of microbial compositions showed that VEGF-C + ADSCs treatment significantly increased the abundance of *g-Lactobacillus* and *g-Bacteroides* but decreased *g-Prevotella* compared with the TNBS group. In addition, more *p-Bacteroidetes* and fewer *p-Firmicutes* were found after VEGF-C stimulation than ADSCs treatment alone. Our data on abundance variation of two phyla were partially inconsistent with some previous studies [63, 64]. This might be due to the difference in the original abundance of flora among the four groups before treatment. These data indicated that the maintenance of gut barrier integrity improved by ADSCs administration and the enhancement of lymphatic drainage retained more beneficial gut microbiota and reduced the harmful microflora produced by TNBS attacks. After ADSCs treatment, LEfSe analysis showed markedly changed several bacterial taxa, including *Ruminococcus*. *Ruminococcus* can adhere to the colonic epithelium, influence intestinal permeability and be involved in the development of strictures in CD patients [65, 66]. Additionally, colonic mucus environments in the mice with chronic colitis and healthy individuals were considered to have microbiota differences, and the mucus microbiome correlates with colitis severity more closely than the faeces or caecum [67]. Therefore, we should further examine the microbial compositions of colon mucus and MLNs to explore the relevant mechanisms.

Our results demonstrated that ADSCs stimulated by VEGF-C were more effective in treating experimental colitis than ADSCs alone, which exhibited great value for CD treatment. However, some limitations exist in our research. Migration of ADSCs to other tissues was not analysed in vivo. The therapeutic effects of VEGF-C-stimulated ADSCs need further comparison with those of medications. Larger samples of faeces and mucus should be collected to detect changes in gut microbiota after treatment. The interaction mechanisms between ADSCs, intestinal inflammation and lymphangiogenesis require in-depth study.

## Conclusion

In summary, this study indicates that ADSCs stimulated by VEGF-C exert stronger effects on the inhibition of inflammation “IN” and the promotion of inflammation “OUT” by enhancing lymph flow drainage combined with increased secretion and immunomodulation of ADSCs. These findings provide theoretical and experimental evidence for novel insights and mechanisms of the intestinal lymphatic system in the treatment of CD by ADSCs.

## Supplementary Information

**Additional file 1: Table 1.** Colitis score. **Table 2.** Disease activity index score parameters. **Table 3.** Scoring system for inflammation-associated macroscopic colonic damage. **Table 4.** Scoring system for inflammation-associated histological changes in TNBS-induced colitis. **Table 5.** Sequences of PCR primers. **Table 6.** Body weight and the number of surviving mice in each experimental group at each time point.

**Additional file 2: Fig. S1.** Homing of ADSCs to the mesenteric lymph nodes (MLNs). The distribution of the injected PKH67-labelled ADSCs (green) in the murine MLNs under a fluorescence microscope with DAPI (blue). Scale bar 100 μm; ×200, magnification.

**Additional file 3: Fig. S2.** mRNA levels of cytokines in the colons. (a) TNF-α, (b) IL-1β, (c) IL-17A and (d) Ang-2.

**Additional file 4: Fig. S3.** T cells in the mesenteric lymph nodes (MLNs). (a) CD4+ T cells, CD8+ T cells in the MLNs were observed by IHC. ×400, magnification. (b) CD4+ T cells in the MLN. (c) CD8+ T cells in the MLN. (N = 3, *P < 0.05, **P < 0.01).

**Additional file 5: Fig. S4.** T cells in the spleens. (a) CD4+ T cells, CD8+ T cells in the spleens were observed by IHC. ×400, magnification. (b) CD4+ T cells in the spleen. (c) CD8+ T cells in the spleen. (N = 3, *P < 0.05, **P < 0.01).

**Additional file 6: Fig. S5.** Diversity and abundance analysis of the faecal microbiota. (a, b) Alpha diversity was calculated using the observed number of Simpson and Shannon indices. (c) PCoA-3D profile of microbial diversity across all samples using Bray-Curtis distances. Each dot represented one sample. (d) The relative abundance of the core microbiota at the phylum level was compared among four groups. (e) The relative abundance of the core microbiota at the genus level was compared among four groups. (N = 3, *P < 0.05, **P < 0.01).

## Data Availability

Additional data and materials that support the findings of current study are available from the corresponding author upon reasonable request.

## Abbreviations

ADSCs: Adipose-derived mesenchymal stem cells
CD: Crohn’s disease
VEGF-C: Vascular endothelial growth factor C
VEGFR-3: Vascular endothelial growth factor receptor 3
LECs: Lymphatic endothelial cells
TNBS: 2,4:,6-Trinitrobenzenesulfonic acid
TGF-β1: Transforming growth factor beta 1
FGF-2: Fibroblast growth factor 2
IGF-1: Insulin-like growth factor 1
Ang-2: Angiopoietin-2
IFN-γ: Interferon gamma
TNF-α: Tumor necrosis factor alpha
IL-1β: Interleukin-1 beta
IL-6: Interleukin-6
IL-10: Interleukin-10
IL-17A: Interleukin-17a
NF-*κ*B: Nuclear factor-*kappa*B
MSCs: Mesenchymal stem cells
BMSCs: Bone marrow mesenchymal stem cells
IBD: Inflammatory bowel disease
GVHD: Graft-versus-host disease
PBS: Phosphate-buffered saline
MSCBM: Mesenchymal stem cell basal medium
ELISA: Enzyme-linked immunosorbent assay
DAI: Disease activity index
H&E: Hematoxylin and eosin
IHC: Immunohistochemical
MLN: Mesenteric lymph nodes
LYVE-1: Lymphatic vessel endothelial hyaluronan receptor-1
LVD: Lymphatic vessel density
MVD: Microvascular density
BSA: Bovine serum albumin
DAB: Diaminobenzidine
qRT-PCR: Quantitative real-time polymerase chain reaction
16S rRNA: 16S ribosomal RNA
ANOVA: Analysis of variance
ASV: Amplicon sequence variant
LDA: Linear discriminant analysis
LEfSe: LDA effect size
PCoA: Principal co-ordinates analysis
ANOVA: Analysis of variance
SD: Standard deviation
ISCs: Intestinal stem cells
Th: T helper
Treg: Regulatory T
DCs: Dendritic cells
