# Supplementary material for: ADSCs stimulated by VEGF-C alleviate intestinal inflammation via dual mechanisms of enhancing lymphatic drainage by a VEGF-C/VEGFR-3-dependent mechanism and inhibiting the NF-κB pathway by the secretome
Source: Stem Cell Res Ther. 2022 Sep 5;13:448. doi: 10.1186/s13287-022-03132-3 (PMC9442958; doi:10.1186/s13287-022-03132-3)
Supplement: Supplementary file 1 — Additional file 1: Table 1. Colitis score. Table 2. Disease activity index score parameters. Table 3. Scoring system for inflammation-associated macroscopic colonic damage. Table 4. Scoring system for inflammation-associated histological changes in TNBS-induced colitis. Table 5. Sequences of PCR primers. Table 6. Body weight and the number of surviving mice in each experimental group at each time point. [file 13287_2022_3132_MOESM1_ESM.docx]

**Supplementary table 1 | Colitis score (scale 0-4)**

| **Score** | **Observation** |
| --- | --- |
| 0 | Normal stool appearance |
| 1 | Slight increase in stool consistency |
| 2 | Moderate increase in stool consistency |
| 3 | Moderate increase in stool consistency and presence of blood in stools |
| 4 | Severe watery diarrhea and gross blooding in stools |

**Supplementary table 2 | Disease activity index score parameters (scale 0-12)**

| **Score** | **Weight loss** | **Stool consistency** | **Bleeding severity** |
| --- | --- | --- | --- |
| 0 | None | Formed and hard | Absence |
| 1 | 1-5% | Formed but soft |  |
| 2 | 6-10% | Soft | Presence |
| 3 | 11-18% | Very soft; wet |  |
| 4 | >18% | Watery diarrhea | Gross |

**Supplementary table 3 | Scoring system for inflammation-associated macroscopic colonic damage (scale 0-8)**

| **Score** | **Observation** | **macroscopic colonic damage** |
| --- | --- | --- |
| 0 | Ulceration | Normal appearance |
| 1 |  | Focal hyperemia, no ulcers |
| 2 |  | Ulceration without hyperemia or bowel well thickening |
| 3 |  | Ulceration with inflammation at one site |
| 4 |  | Two or more sites of ulceration and inflammation |
| 5 |  | Major sites of damage extending > 1 cm along length of colon |
| 0 | Adhesions | No adhesions |
| 1 |  | Minor adhesions, colon can be easily separated from the other tissues |
| 2 |  | Major adhesions |
| 0.5-1 | Thickness | Weight/length ratio was calculated to estimate colon thickness |

**Supplementary table 4 | Scoring system for inflammation-associated histological changes in TNBS-induced colitis (scale 0-4)**

| **Score** | **Histologic changes in TNBS-induced colitis** |
| --- | --- |
| 0 | No evidence of inflammation |
| 1 | Low level of leukocyte infiltration with infiltration seen in <10% HPF. No structural changes. |
| 2 | Moderate leukocyte infiltration with infiltration seen in 10% to 25% HPF, crypt elongation, bowel wall thickening that does not extend beyond the mucosa layer, and no ulcerations. |
| 3 | High level of leukocyte infiltration seen in 25% to 50% HPF, crypt elongation, infiltration beyond the mucosal layer, thickening of the bowel wall and superficial ulcerations. |
| 4 | Marked degree of transmural leukocyte infiltration seen in >50% HPF, elongated and distorted crypts, bowel-wall thickening, and extensive ulcerations. |

**Supplementary table 5 | Sequences of PCR primers**

| **Gene** | **Forward primer (5'--3')** | | **Reverse primer (5'--3')** | |
| --- | --- | --- | --- | --- |
| Human-GAPDH | GGAGCGAGATCCCTCCAAAAT | GGCTGTTGTCATACTTCTCATGG | |  |
| Human-VEGF-C | GAGGAGCAGTTACGGTCTGTG | TCCTTTCCTTAGCTGACACTTGT | |  |
| Human-VEGFR-3 | TGCACGAGGTACATGCCAAC | GCTGCTCAAAGTCTCTCACGAA | |  |
| Mouse-GAPDH | AGGTCGGTGTGAACGGATTTG | TGTAGACCATGTAGTTGAGGTCA | |  |
| Mouse-IFN-γ | CAATGAACGCTACACACTGC | ATGTCACCATCCTTTTGCCAG | |  |
| Mouse-TNF-α | CATCTTCTCAAAATTCGAGTGACAA | TGGGAGTAGACAAGGTACAACCC | |  |
| Mouse-IL-1β | GAGGACATGAGCACCTTCTTT | GCCTGTAGTGCAGTTGTCTAA | |  |
| Mouse-IL-6 | TCTATACCACTTCACAAGTCGGA | GAATTGCCATTGCACAACTCTTT | |  |
| Mouse-IL-10 | CAGCCGGGAAGACAATAACTG | CCGCAGCTCTAGGAGCATGT | |  |
| Mouse-IL-17A | TCAAAGCTCAGCGTGTCCAA | TCTTCATTGCGGTGGAGAGTC | |  |
| Mouse-TGF-β1 | CCACCTGCAAGACCATCGAC | CTGGCGAGCCTTAGTTTGGAC | |  |
| Mouse-FGF-2 | TGGTGACCACAAGCTGAATG | TCCCTTGATAGACACAACTCCTC | |  |
| Mouse-IGF-1 | CACATCATGTCGTCTTCACACC | GGAAGCAACACTCATCCACAATG | |  |
| Mouse-Ang-2 | GGACAGTCATCCAACACCGAG | GACTCTTCACCAGCGAGGTAG | |  |
| Mouse-VEGF-C | GAGGTCAAGGCTTTTGAAGGC | CTGTCCTGGTATTGAGGGTGG | |  |
| Mouse-VEGFR-3 | ACAGAAGCTAGGCCCTACTG | ACCCACATCGAGTCCTTCCT | |  |

**Supplementary table 6 | Body weight and the number of surviving mice in each experimental group at each time point**

|  | Control | TNBS | ADSCs | VEGF-C+ADSCs |
| --- | --- | --- | --- | --- |
| Day 1 | 1.64±1.33 (N=9) | -6.36±6.07 (N=9) | -1.12±3.10 (N=9) | -2.37±2.62 (N=9) |
| Day 8 | 5.63±2.97 (N=9) | -8.19±6.38 (N=7) | -3.59±2.42 (N=9) | 3.37±3.99 (N=9) |
| Day 15 | 9.76±3.97 (N=9) | -1.13±5.02 (N=6) | -0.50±6.69 (N=9) | 6.92±5.18 (N=9) |
| Day 22 | 10.02±3.56 (N=9) | 0.05±7.42 (N=6) | 2.39±8.07 (N=9) | 7.99±7.41 (N=9) |
| Day 29 | 12.24±3.45 (N=9) | 4.14±4.80 (N=5) | 5.19±12.51 (N=6) | 11.41±11.65 (N=8) |
| Day 35 | 15.39±3.60 (N=9) | 9.06±3.28 (N=5) | 10.95±7.94 (N=6) | 13.39±9.50 (N=8) |
