# Supplementary figures and images for: ADSCs stimulated by VEGF-C alleviate intestinal inflammation via dual mechanisms of enhancing lymphatic drainage by a VEGF-C/VEGFR-3-dependent mechanism and inhibiting the NF-κB pathway by the secretome
Source: Stem Cell Res Ther. 2022 Sep 5;13:448. doi: 10.1186/s13287-022-03132-3 (PMC9442958; doi:10.1186/s13287-022-03132-3)

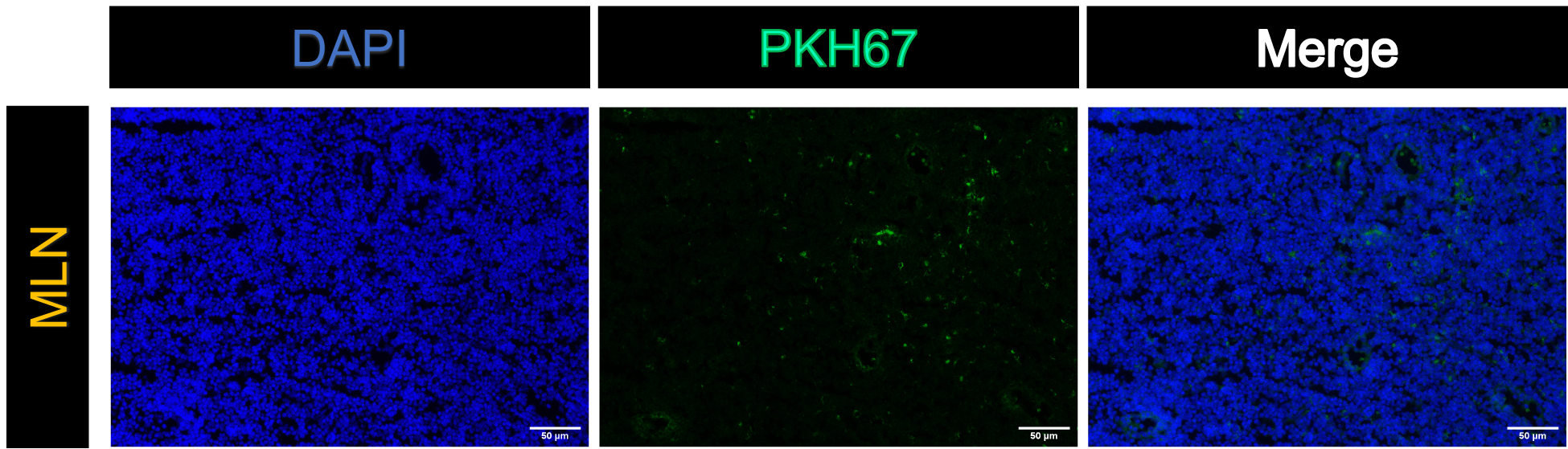

Supplement: Supplementary file 2 — Additional file 2: Fig. S1. Homing of ADSCs to the mesenteric lymph nodes (MLNs). The distribution of the injected PKH67-labelled ADSCs (green) in the murine MLNs under a fluorescence microscope with DAPI (blue). Scale bar 100 μm; ×200, magnification. [file 13287_2022_3132_MOESM2_ESM.tif]

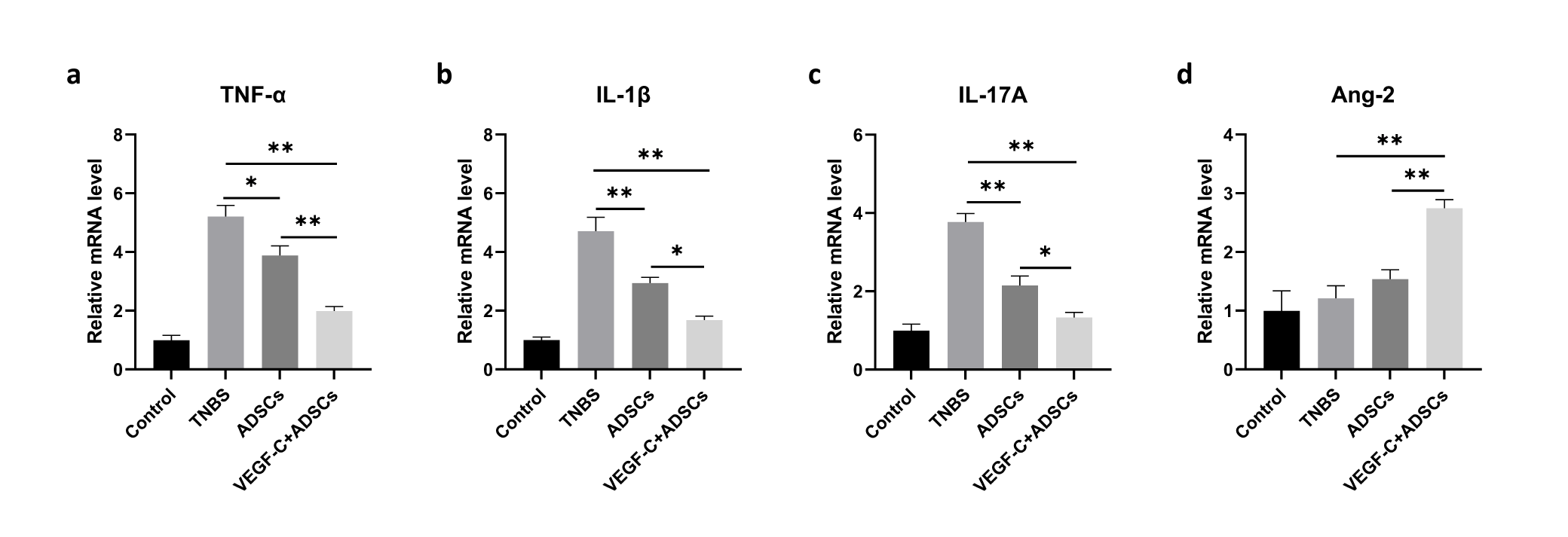

Supplement: Supplementary file 3 — Additional file 3: Fig. S2. mRNA levels of cytokines in the colons. (a) TNF-α, (b) IL-1β, (c) IL-17A and (d) Ang-2. [file 13287_2022_3132_MOESM3_ESM.tif]

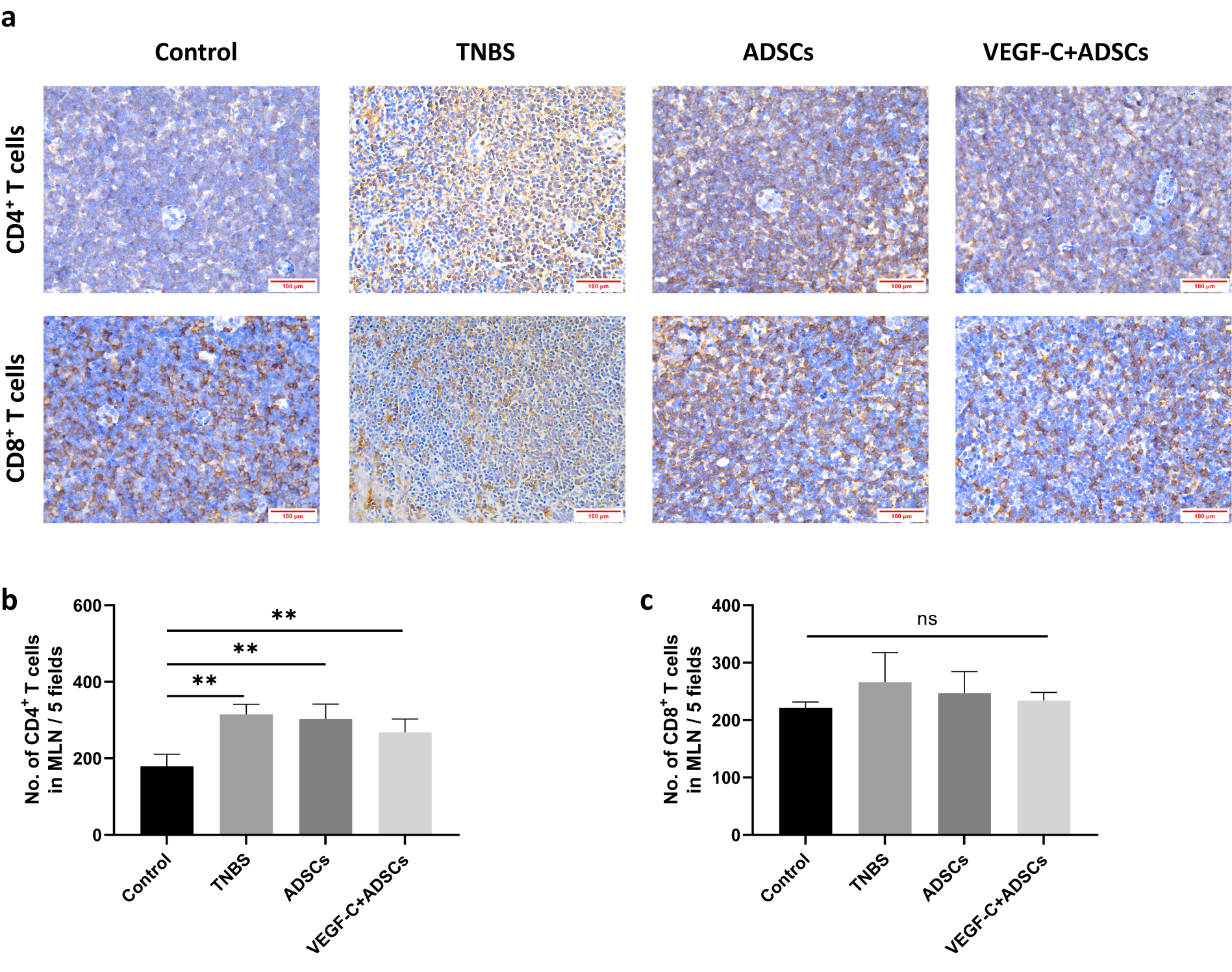

Supplement: Supplementary file 4 — Additional file 4: Fig. S3. T cells in the mesenteric lymph nodes (MLNs). (a) CD4+ T cells, CD8+ T cells in the MLNs were observed by IHC. ×400, magnification. (b) CD4+ T cells in the MLN. (c) CD8+ T cells in the MLN. (N = 3, *P < 0.05, **P < 0.01). [file 13287_2022_3132_MOESM4_ESM.tif]

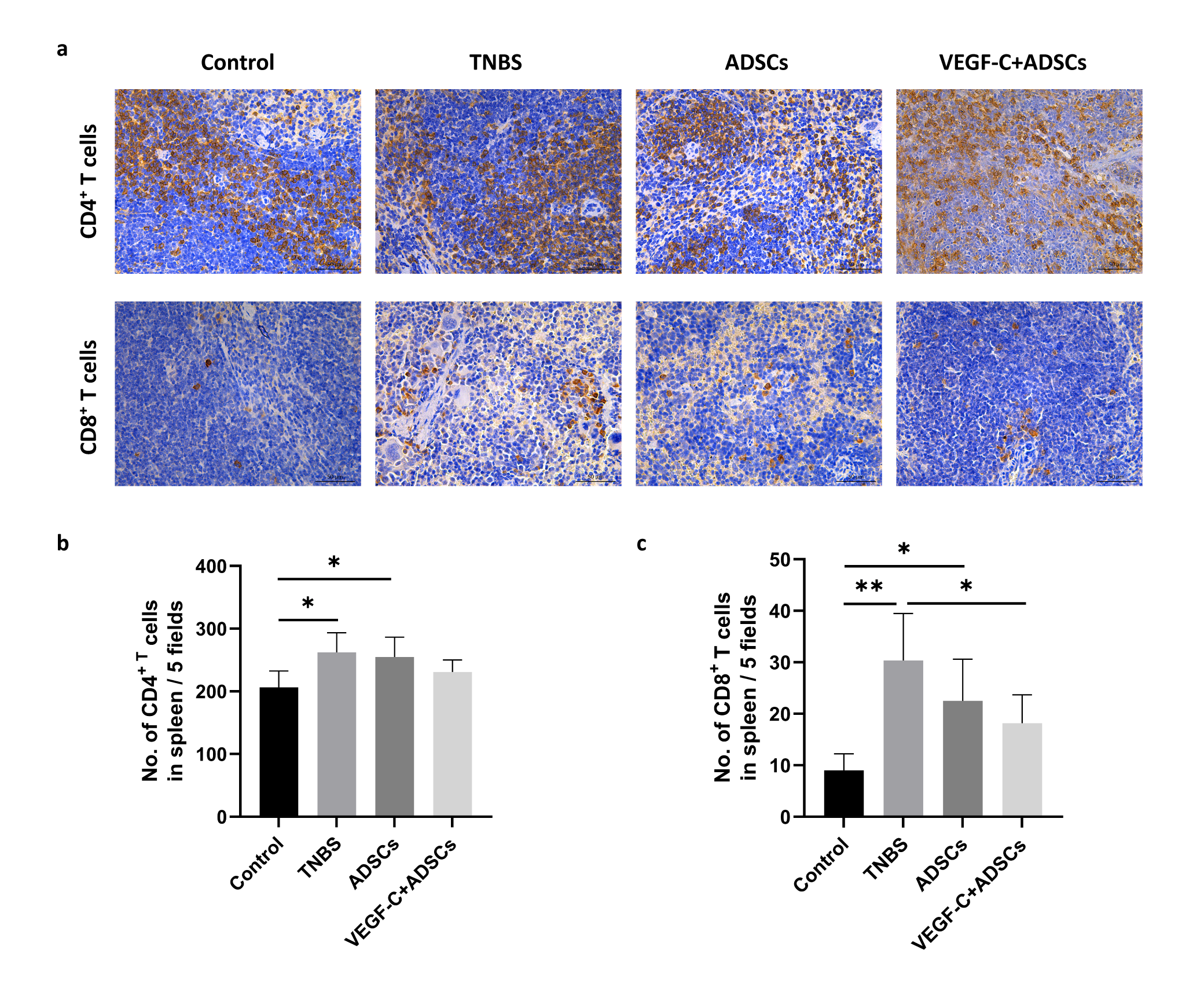

Supplement: Supplementary file 5 — Additional file 5: Fig. S4. T cells in the spleens. (a) CD4+ T cells, CD8+ T cells in the spleens were observed by IHC. ×400, magnification. (b) CD4+ T cells in the spleen. (c) CD8+ T cells in the spleen. (N = 3, *P < 0.05, **P < 0.01). [file 13287_2022_3132_MOESM5_ESM.tif]

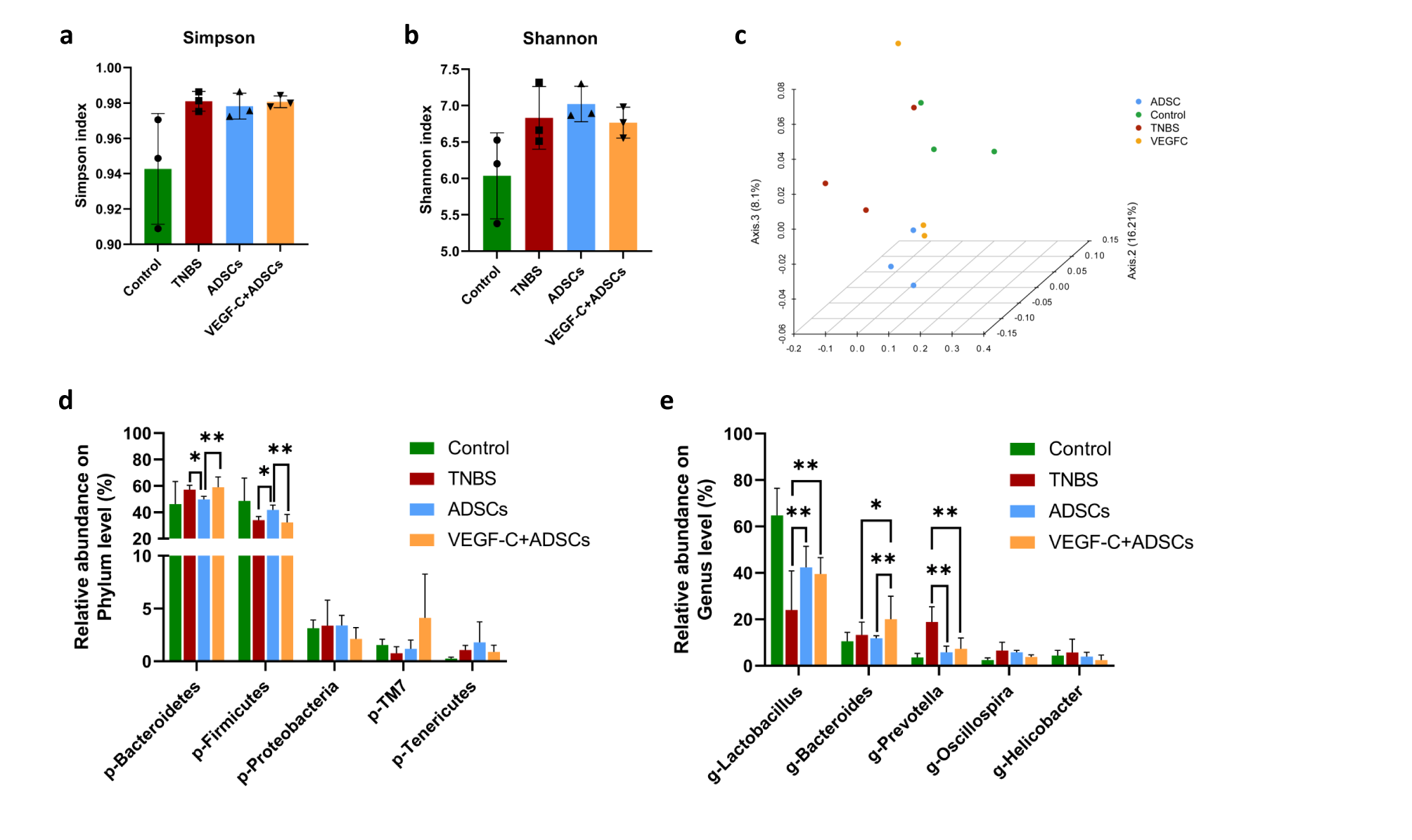

Supplement: Supplementary file 6 — Additional file 6: Fig. S5. Diversity and abundance analysis of the faecal microbiota. (a, b) Alpha diversity was calculated using the observed number of Simpson and Shannon indices. (c) PCoA-3D profile of microbial diversity across all samples using Bray-Curtis distances. Each dot represented one sample. (d) The relative abundance of the core microbiota at the phylum level was compared among four groups. (e) The relative abundance of the core microbiota at the genus level was compared among four groups. (N = 3, *P < 0.05, **P < 0.01). [file 13287_2022_3132_MOESM6_ESM.tif]
